# SREBP-1 inhibitor Betulin enhances the antitumor effect of Sorafenib on hepatocellular carcinoma via restricting cellular glycolytic activity

**DOI:** 10.1038/s41419-019-1884-7

**Published:** 2019-09-11

**Authors:** Fan Yin, Fan Feng, Lei Wang, Xiaoning Wang, Zongwei Li, Yu Cao

**Affiliations:** 1 Department of Oncology, the Second Medical Centre & National Clinical Research Center of Geriatric Disease, Chinese PLA General Hospital, 100853 Beijing, People’s Republic of China; 2 Center for Clinical Laboratory, the Fifth Medical Centre, Chinese PLA General Hospital, 100039 Beijing, People’s Republic of China; 3 Department of Gastroenterology, the First Medical Centre, Chinese PLA General Hospital, 100843 Beijing, People’s Republic of China; 4 Department of Blood Transfusion, the First Hospital of Jilin University, Changchun, 130021 Jilin Province People’s Republic of China; 5 Department of Immunology, H. Lee Moffitt Cancer Center & Research Institute, 33612 Tampa, FL USA

**Keywords:** Biochemistry, Cancer, Cell biology

## Abstract

Lipid metabolism that correlates tightly to the glucose metabolic regulation in malignant cells includes hepatocellular carcinoma (HCC) cells. The transcription factor Sterol Regulatory Element Binding Protein 1 (SREBP-1), a regulator of fatty acid synthesis, has been shown to pivotally regulate the proliferation and metastasis of HCC cells. However, the intrinsic mechanism by which SREBP-1 regulates the survival of HCC cells remains unclear. In this study, among HCC patients who had dismal responses to Sorafenib, a high SREBP-1 level was found in the tumors and correlated to poor survival. This observation suggested the negative role of SREBP-1 in clinical HCC prognosis. Our mechanistical studies reveal that the inhibition of SREBP-1 via its inhibitor Betulin suppresses cellular glucose metabolism. In addition to the reduced glycolytic activity, a thwarted metastatic potential was observed in HCC cells upon Betulin administration. Moreover, our data show that SREBP-1 inhibition facilitated the antitumor effects of Sorafenib on HCC cells and xenograft tumors.

## Introduction

In China, the population of chronic asymptomatic Hepatitis B Virus (HBV) infection-associated patients exceeds 80 million^1–3^. Hepatocellular carcinoma (HCC) is the final malignant disease developed from liver lesions such as HBV infection; it is a fatal threat to life and an increasing burden to the public medical service^4–6^. Thus, it is urgent to develop effective therapeutic approaches for HCC. At present, kinase inhibitors represented by Sorafenib are the major treatment choice for advanced HCC patients^7,8^. As an oral administrative multi-target protein-kinase inhibitor, Sorafenib downregulates the activity of RTKs (receptor protein tyrosine kinases include vascular endothelial growth factor receptor 2/3 [VEGFR-2/3], the hepatocyte factor receptor [c-Kit], Fms-like tyrosine kinase [FLT-3], and platelet-derived growth factor receptor-β [PDGFR-β]) and inhibits the proliferation, metastasis, and angiogenesis of HCC cells^9–11^. The randomized controlled phase III trials, represented by the SHARP (Sorafenib HCC Assessment Randomized Protocol) clinical trial, have shown that Sorafenib significantly prolongs the median survival of patients compared with the placebo group, making it a first-line antitumor agent for advanced HCC treatment^12–15^. However, the sensitivity of Sorafenib in HCC patients varies, with a large proportion finally developed for Sorafenib resistance unexpectedly^16,17^. Thus, it is of great importance to discover a new drug and/or to develop a novel therapeutic strategy that increases the efficacy of Sorafenib.

Cellular metabolisms related to ATP production in malignant cells are more active than normal cells^18^. HCC cells have exaggerated glucose uptake capability for their intensive anaerobic glycolysis which provides them enough energy to survive in harsh tumor microenvironment^19,20^. The chemoresistance of HCC cells closely correlates to the cellular metabolism; through the overwhelming anaerobic glycolytic activity, the accommodation of metabolites and the enhanced epithelial–mesenchymal transition (EMT) afterward take place^21,22^. Thus, glucose metabolism is a potential target of developing antagonistic therapy for chemoresistance.

It is known that 60% of the carbons in glucose are used for fatty acid synthesis, and lipid metabolism also plays vital roles in regulating glucose uptake and glycolytic activities^23^. SREBP-1 is a core transcription factor in lipid metabolism; it induces the transcription of a series of genes involved in fatty acid and triglyceride synthesis^24^. Inhibition of the activity of SREBP-1 decreased the synthesis and accommodation of fat and impeded the glucose uptake^25–27^. Betulin belongs to lupane-type pentacyclic triterpenoids, which are widely found in plants such as Pulsatilla chinensis (Bunge) Regel; they have multiple biological functions, including antitumor activity^28–30^. Betulin directly binds to the SCAP (SREBP cleavage-activating protein) to inhibit the cleavage and activation of SREBP-1^31,32^. In the present study, we performed gene expression analysis on HCC patients after Sorafenib treatment, and found that the high SREBP-1 expression correlates to a poor clinical outcome. Mechanistically, our results indicate that SREBP-1 inhibition represses the cellular glycolytic activity of HCC cells, and restricts the metastasis of those malignant cells both in vitro and in vivo. We also found that Betulin works synergistically with Sorafenib on s.c. and in situ HCC tumors. Our study suggests that the SREBP-1 inhibitor and Sorafenib combination can be a novel therapeutic option for advanced HCC treatment.

## Materials and methods

### Cell lines and reagents

The hepatic cell lines: non-tumor cell line L-02, HCC cell lines HepG2, Hu7, SMMC-7721, and BEL-7402, a lowly aggressive HCC cell line (MHCC97-L), or highly aggressive HCC cell lines (malignant cells) MHCC97-H was purchased from Type Culture Collection of the Chinese Academy of sciences (Shanghai, P.R. China). Cells were cultured in DMEM (Dulbecco’s Modified Eagle Medium) with 10% fetal bovine serum (FBS). The cDNA samples derived from HCC clinical specimens were gifts from Dr. Fan Feng in No. 302nd hospital, Chinese PLA. The HCC clinical specimens were collected and obtained with the informed consent of patients and with approval for experiments from No. 302nd hospital, Chinese PLA. A total of 52 HCC cases were included (Supplementary Table 1)^33,34^. Antitumor agents: Betulin was purchased from MCE Corporate (NJ, USA, Cat. No. HY-N0083); Sorafenib was purchased from Selleck Corporation (Houston, TX, USA, Cat. No. S7397). The expression vector of SREBP-1 and SREBP-1 siRNA was purchased from Vigene Corporation, Jinan, China. The siRNA sequence of SREBP-1 is 5ʹ-GCUCCUCACUUGAAGGCUUTT-3ʹ. A lentivirus particle of siSREBP-1 was prepared by Vigene Corporation, Jinan, China.

### Real-time quantitative PCR

After treating the cells with a series of concentrations of Betulin or Sorafenib (Supplementary Table 2), the RNA samples of the cells were collected and reverse transcribed into cDNA according to the manufacturer’s instructions (Thermo Fisher Scientific, Waltham, MA, USA). For cDNA samples derived from clinical specimens and cells, quantitative PCR assays were performed according to the manufacturer’s instructions (Thermo Fisher Scientific, Waltham, MA, USA) and methods described by Liang et al. and Ji et al.^35,36^. For primers used in quantitative PCR detection, see Supplementary Table 3.

### Western blot experiments

For cell-based experiments, L-02, HepG2, MHCC97-H, MHCC97-L, BEL-7402, SMMC-7721, and Hu7 cells were cultured and harvested after the indicated times. For animal experiments, tumor tissues were harvested and digested. Protein samples were extracted from cells and analyzed for SDS-PAGE. Then, protein samples were *trans*-printed into PVDF membranes. Next, the membranes were blocked by 5% BSA diluted with TBST at 37 °C for 2 h. After blocking, the membranes were in turn incubated by primary antibodies (anti-SREBP-1 antibody [Santa Cruz, USA] or anti-β-Actin antibody [Abcam Corporation, UK]) and the secondary antibody (HRP-coupling antibody, Abcam Corporation, UK). The antibodies of PARP or ki67 were described in our previous work. Membrane exposure was performed in chemiluminescence by using an ECL kit (Amersham Biosciences, Piscataway, NJ, USA)^37,38^.

### MTT assay

After treating HCC cells with the indicated concentrations of agents (Supplementary Table 3), the MTT assay was performed. The amount of HCC cells was determined by the absorbance values of the cell samples at a wavelength of 490 nm. On this basis, the inhibition rate of the drug action was calculated by the OD values. The calculation formula of the inhibition rate is (absorbance value at 490 nm in the control group−absorbance at 490 nm in the experimental group)/ absorbance at the wavelength of 490 nm in the control group × 100%^39–41^.

### In vitro cell migration and invasion

After the HCC cells were transfected with the expression vectors or treated with the indicated concentration of agents, the cells were collected for Transwell experiments: the Transwell chamber was pre-plated with ECM glue, and the cells were added to the chamber, fixed for 12–16 h, stained, and photographed. For the obtained photographs, quantitative analysis was performed by using image analysis software Image J^42,43^. The specific calculation formula is relative invasion/migration cell number = cell total pixel/image total pixel; drug inhibition rate = (control group relative invasion/migration cell number–experiment group) / (control group relative invasion/migration cell number) × 100%.

### Metabolic examinations

Metabolic examinations were carried out according to the methods provided by Li et al.^44,45^. Glycolytic activity examinations were performed in either cultured tumor cells or cells isolated from tumors. The glucose uptake (Glucose Uptake Assay Kit (Colorimetric), (ab136955), Abcam), lactate (Lactate-Glo™ Assay Kit, Promega), and ATP (ATP Assay Kit (Colorimetric/Fluorometric) (ab83355), Abcam) production, LDH activity (Lactate Dehydrogenase Activity Assay Kit, Cat#: MAK066, Sigma), extracellular acidification rate (ECAR, Seahorse XF Glycolysis Stress Test Kit, Agilent), and oxygen-consumption rate (OCR, Seahorse XF Cell Mito Stress Test Kit, Agilent) were measured according to the manufacturer's instructions. On this basis, the inhibition rate was calculated: (control group biochemical index–experimental group biochemical index)/(control group biochemical index) × 100%.

### Subcutaneous HCC tumor model

All animal experiments and protocols were approved by the Animal Care and Use Committee of the General hospital, Chinese PLA, and all animal experiments were carried out in accordance with the UK Animals (Scientific Procedures) Act, 1986 and its associated guidelines. The 4–6-week-old nude mice (Bal B/c mice with T cell/thymus deletion features) were used. HCC cells were cultured, and the cells were injected subcutaneously into nude mice. Then, antitumor agents were intragastrically administered 2–3 days after injection of HCC cells^46,47^. The concentration gradient of the agents used in the subcutaneous tumor formation experiments is shown in Supplementary Table 4. Animals were intragastrically administered with different concentrations of Betulin, Sorafenib, or Betulin + Sorafenib. The drug was administered once per 2 days, and after 10 times’ treatments (21 days), the animals were killed to collect tumor tissue. The tumor size was calculated and the tumor was weighed. The inhibition rate of the drug acting on the subcutaneous tumor formation of HCC cells was calculated according to tumor size and tumor weight: the inhibition rate calculated based on tumor size [(control tumor size)−(tumor size of the drug treatment group)]/(control tumor size) × 100%; the inhibition rate calculated based on tumor weight [(control tumor weight)−(tumor weight of the drug treatment group)]/(control group tumor weight) × 100%.

### Intrahepatic tumor models in nude mice

The collected HCC subcutaneous tumors were prepared into tissue microblocks, and the tissue microblocks were transplanted into the liver of the nude mice^48,49^. After 2–3 days of tumor transplantation, oral administration of agents was performed, and a solvent control, Betulin or Sorafenib was administered. After 4–6 weeks, the animals were imaged in vivo. Nude mice were inhaled with isoflurane, and the nude mice were injected with 200 μCi (7.4 MBq) of the ^18^F-FDG radionuclide probe via the tail-vein injection, and the PET testing of animal was performed after about 40–50 min. According to the results of PET, the animal's anatomy was collected to obtain liver specimens. The specimen was photographed, and the image analysis software Image J was used to analyze the image to determine the relative area of the tumor lesions: [(the total number of pixels in the tumor lesion)/(the total number of pixels in the image)]/[(the total number of pixels in the liver lesion)/(total number of pixels in the image)] × 100%; using the Geiger counter to analyze the intensity of the specimen, the liver nuclide intensity is (unit weight of liver organ nuclides)/(unit weight of blood nuclides). The inhibition rate calculated based on the relative area of the tumor lesions [(relative area of tumor lesions in the control group) − (relative area of tumor lesions in the drug treatment group)]/(relative area of tumor lesions in the control group) × 100%; based on tumor weight, the calculated inhibition rate was [(control liver nuclide intensity)–(hepatic nuclide intensity in the drug treatment group)]/(control liver nucleus intensity) × 100%^50–52^.

### Statistical analysis

Statistical analysis was performed by Bonferroni’s correction with or without two-way ANOVA by using SPSS statistical software (IBM Corporation, Armonk, NY, USA). The IC_50_ and EC_50_ value of each agent was calculated by the Origin (Origin 6.1; OriginLab Corporation, Northampton, MA, USA). A *P*-value of <0.05 was considered statistically significant.

## Results

### SREBP-1 level negatively correlates to the prognosis of HCC patients

**Fig. 1:**
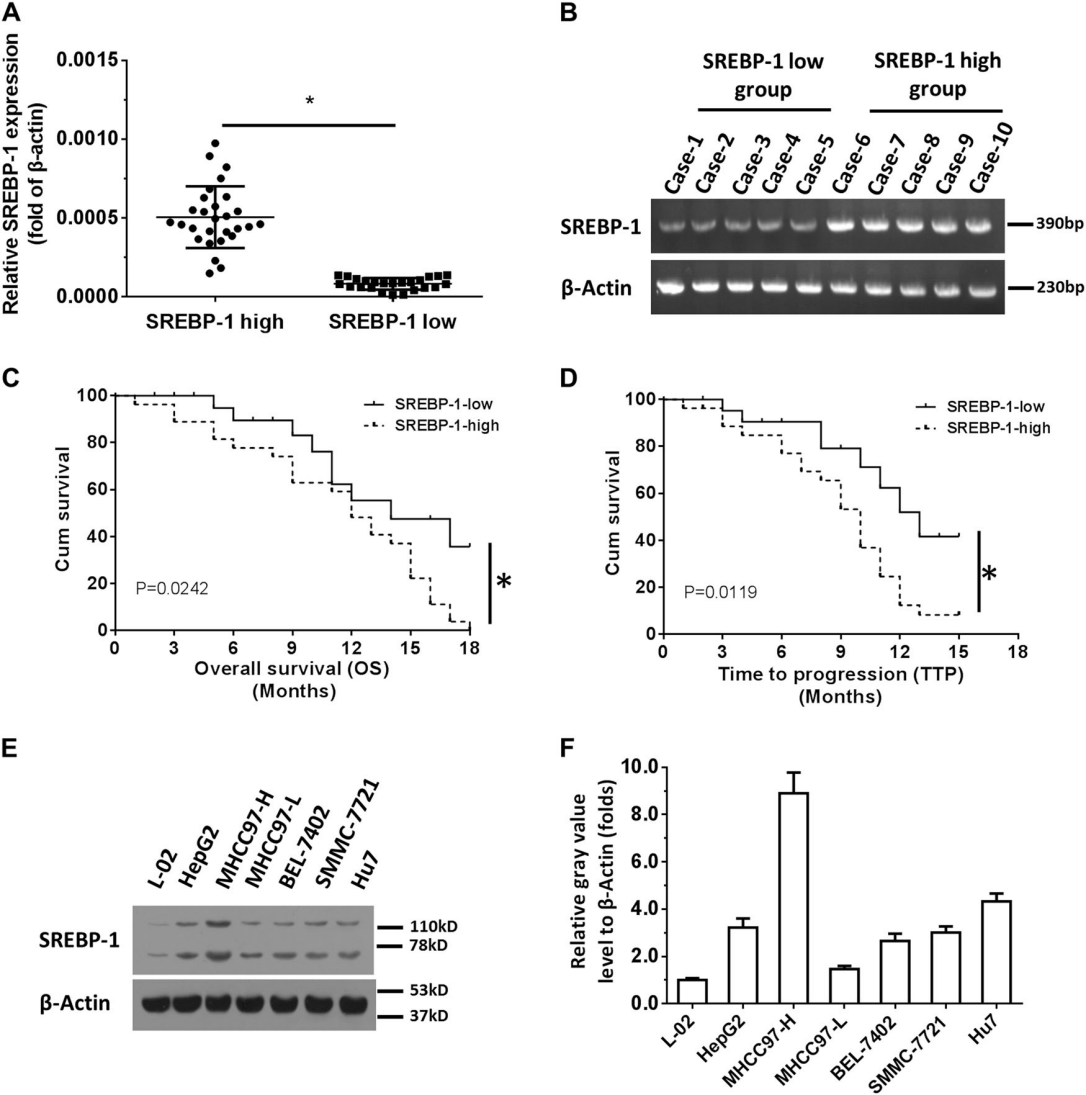
High SREBP-1 level correlates to poor prognosis of advanced HCC patients who received Sorafenib treatment. **a** Patients were divided into SREBP-1 high and SREBP-1 low groups based on the expression level. **b** PCR results from ten representative specimens (five high and five low). **c** Overall survival (OS) comparison of HCC patients in the SREBP-1 high group and the SREBP-1 low group. **d** Time to progress (TTP) of HCC patients in the SREBP-1 high group and the SREBP-1 low group. **e**, **f** The relative SREBP-1 expressions at both mRNA (**e**) and protein levels (**f**) in different cell lines. Survival analysis was performed by the Kaplan–Meier method and compared by the log-rank test. Paired samples were tested by the paired-sample *t*-test. Significant: *p < 0.05 in all figures

The expression of SREBP-1 in 52 fine-needle aspiration tumor specimens derived from HCC patients who received Sorafenib treatment was measured and analyzed. Then these patients were divided into two groups according to the median value of the SREBP-1 expression: high and low SREBP-1 groups (Fig. 1a, b). After analysis, we found that the prognosis of patients with low SREBP-1 levels was better than that of patients with high SREBP-1 levels (Fig. 1c, d). The time to progression (TTP) and the overall survival (OS) time of the high SREBP-1 group were shorter compared with those of the low SREBP-1 group (Table 1), suggesting that SREBP-1 negatively correlates to HCC treatment outcomes. In addition, we found that the level of SREBP-1 in HCC cells was significantly higher than that of L-02 nonmalignant liver cells (Fig. 1e, f). High aggressive MHCC97-H cells have the highest SREBP-1 expression among the tested HCC cell lines, suggesting that SREBP-1 correlates with a higher metastatic capacity.

**Table 1:** SREBP-1 expression and outcome of sorafenib treatment

	SREBP-1 mRNA expression		*P*
	High (*n* = 27)	Low (*n* = 25)	
TTP	9.1	11.8	0.012
	7.7–10.4 (M)	10.1–13.5 (M)	
OS	11.3	13.7	0.024
	9.4–13.1 (M)	11.6–15.8 (M)	
Overall response rate (CR + PR)	1 (3.7%)	3 (12%)	
Disease control rate (CR + PR + SD)	5 (18.5%)	8 (32%)	

*TTP* time to progress, *OS* overall survival, *PR* partial remission, *CR* complete remission, *SD* stable disease, *M* months

### SREBP-1 promotes HCC cell proliferation and metastasis

We further tested the role of SREBP-1 in the proliferation and metastasis of HCC cells. Knockdown of SREBP-1 expression in MHCC97-H cells led to an inhibited proliferation and metastasis (Supplementary Fig. 1). Accordingly, SREBP-1 overexpression in MHCC97-L cells, which has the lowest SREBP-1 expression level among the tested HCC cell lines, promoted cell proliferation and metastasis (Supplementary Fig. 2). Similarly, SREBP-1 inhibition through its inhibitor Betulin in MHCC97-H cells mimicked the effects of gene knockdown (Supplementary Fig. 3A–C). To further verify the specificity of Betulin, we constructed a luciferase reporter gene vector which harbored a SREBP-1-binding element, transfected MHCC97-H cells with the reporter vectors, and performed Betulin or vehicle administration. We found that Betulin treatment decreased the luciferase activity in a dose-dependent manner, compared with the vehicle control (Supplementary Fig. 3D). Taken together, these results validate that SREBP-1 promotes HCC cell proliferation and metastasis, and the SREBP-1 inhibitor Betulin blocks SREBP-1's transcription factor activity specifically.

### Knockdown or inhibition of SREBP-1 thwarts the glycolytic activity of HCC cells

**Fig. 2:**
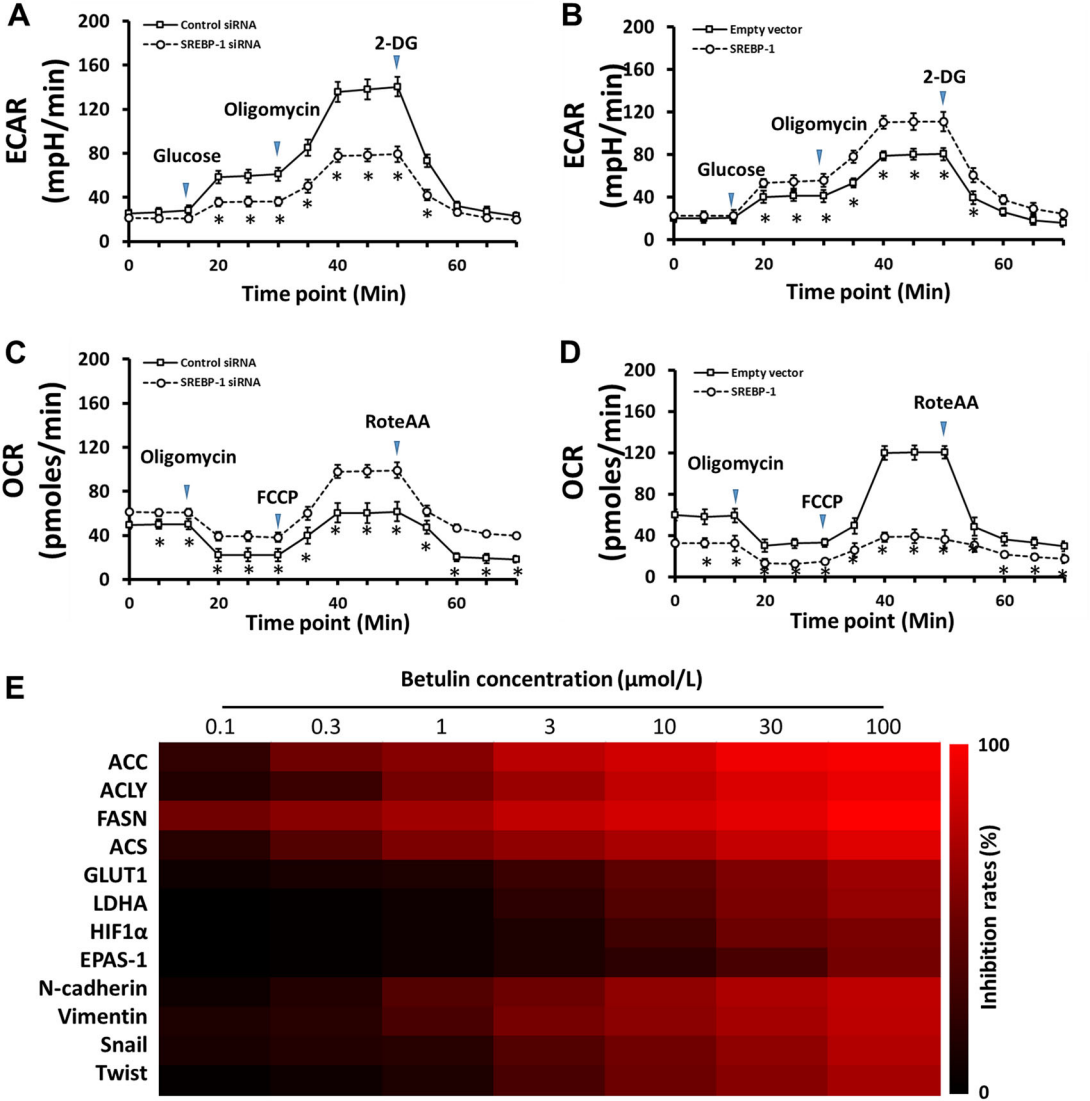
SREBP-1 regulates the glycolytic activity of HCC cells. **a** Extracellular acidification rate (ECAR) measurement in high metastatic MHCC97-H cells transfected with control or SREBP-1 siRNAs. **b** ECAR measurement in low metastatic MHCC97-L cells transfected with empty or SREBP-1-expressing vectors. **c** Oxygen-consumption rate (OCR) measurement in MHCC97-H cells from **a**. **d** OCR measurement in MHCC97-L cells from **c**. **e** MHCC97-H cells were treated with the indicated concentrations of Betulin (100, 30, 10, 3, 1, 0.3, or 0.1 μmol/L). Next, the cells were harvested for quantitative RT-PCR. The inhibition rates of Betulin on gene expression were calculated and shown by a heatmap

Next, we tested the role of SREBP-1 in the regulation of glycolytic activity of HCC cells. Knockdown of SREBP-1 by siRNA decreased glucose uptake and lactate dehydrogenase (LDH) activity in MHCC97-H cells (Supplementary Fig. 4A, B), suggesting that SREBP-1 downregulation impairs anaerobic glycolytic activity. Accordingly, reduced ATP and lactate productions were found upon SREBP-1 knockdown (Supplementary Fig. 4C, D). Moreover, in the SREBP-1-overexpressed MHCC97-L cells, we detected higher glucose uptake, increased LDH activity, and more lactate and ATP production (Supplementary Fig. 4E–H). Next, the glycolysis stress test showed that the SREBP-1 knockdown results in the decreased extracellular acidification rate (ECAR), indicating a lower overall glycolytic activity (Fig. 2a). Similarly, SREBP-1 overexpression induced a higher ECAR in MHCC97-L cells (Fig. 2b), suggesting the regulatory role of SREBP-1 on HCC cell glycolysis. As an opposite oxidative phosphorylation activity is often observed upon alterations of glycolysis occurrence in tumor cells, which was also termed as the Warburg effect, we performed mitochondrial respiration tests for the oxygen-consumption rate (OCR) measurement. Our results showed increased OCR in SREBP-1 knockdown, whereas decreased OCR in SREBP-1 overexpression groups (Fig. 2c, d). Administration of the SREBP-1 inhibitor Betulin on MHCC97-H cells showed the similar effects, compared with the SREBP-1 knockdown, on lactate and ATP production, and glycolytic activity (Supplementary Fig. 5). Taken together, these data suggest that knockdown or inhibition of SREBP-1 dampens the glucose uptake, anaerobic glycolytic activity, and ATP production of HCC cells.

By testing the transcription level of the key genes in metabolism pathways, we found that Betulin treatment reduced the expression of lipid metabolism-associated genes, including acetyl-CoA carboxylation (ACC), ATP citrate lyase (ACLY), fatty acid synthase (FASN), and acyl-CoA synthetase (ACS) as expected (Fig. 2e, Supplementary Fig. 6 and Supplementary Table 5). For glucose metabolism, glucose uptake, and hypoxia stress, we found decreased expression of GLUT1, LDHA, HIF-1α, and EPAS-1 genes, which is in line with our discovery in the present work. Moreover, given that an abnormal Warburg effect in malignant cells may alter the EMT, which induces the resistance of HCC cells to antitumor chemotherapies, we tested EMT marker genes Twist, Snail, N-cadherin, and Vimentin, and found that they were downregulated after Betulin treatment, indicating a correlation between SREBP-1 and chemoresistance.

### SREBP-1 enhances the sensitivity of HCC cells to Sorafenib

**Fig. 3:**
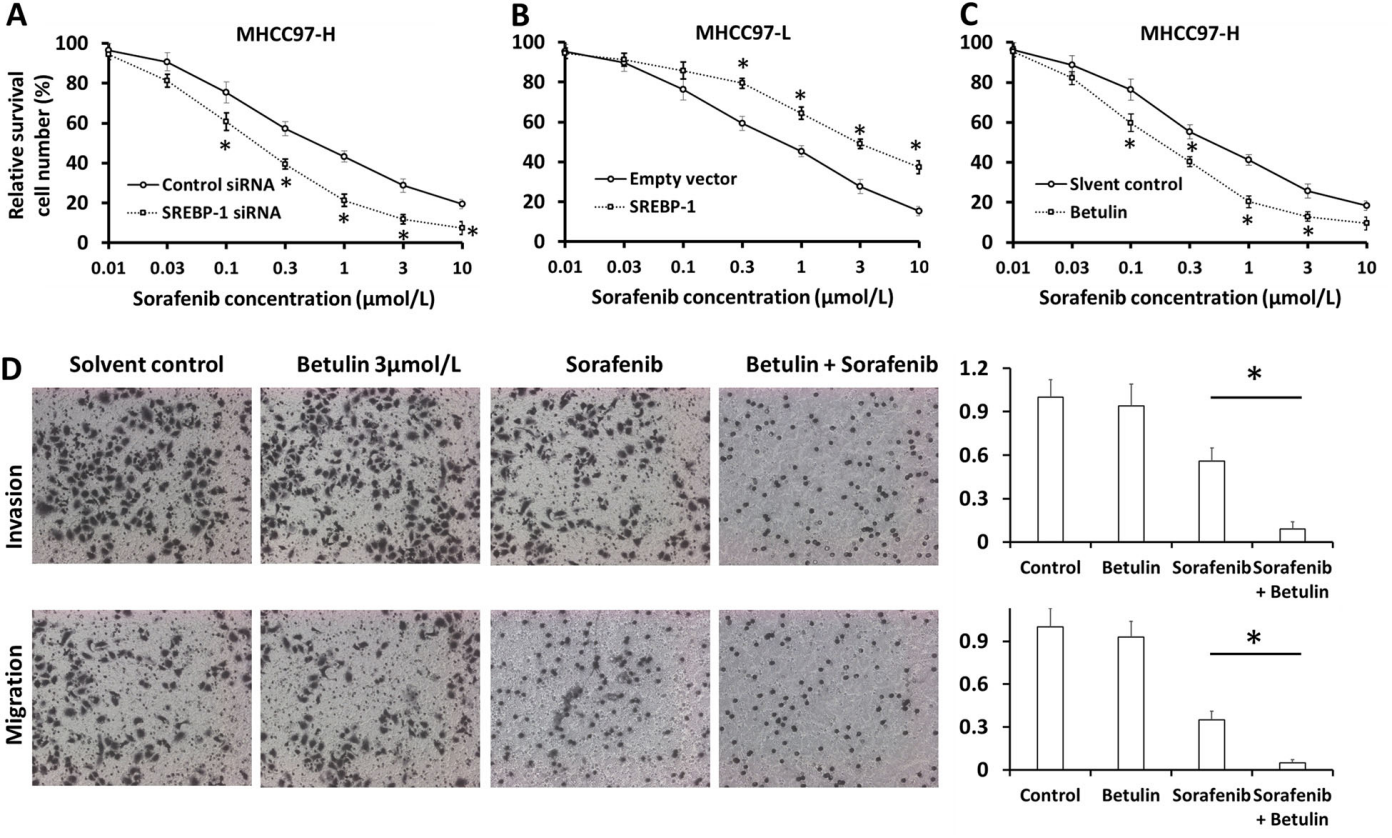
SREBP-1 regulates the sensitivity of HCC cells to Sorafenib. **a** MHCC97-H cells transfected with control or SREBP-1 siRNAs were treated with the indicated concentrations of Sorafenib for 48 h and then harvested for MTT experiments. All the results were shown as mean ± SD. **b** MHCC97-L cells transfected with empty or SREBP-1-expressing vectors were treated with the indicated concentrations of Sorafenib for 48 h and then harvested for MTT experiments. **c** MHCC97-H cells pretreated with 3 μM Betulin or vehicle control were treated with the indicated concentrations of Sorafenib for 48 h and then harvested for MTT experiments. **d** MHCC97-H cells pretreated with 3 μmol/L Betulin or vehicle control were treated with Sorafenib at the IC_50_ concentration for 48 h. Then, cells were harvested for transwell experiments

To study the role of SREBP-1 in chemoresistance, we performed SREBP-1 overexpression, knockdown, or inhibition in HCC cells in the presence of Sorafenib treatment. Silence of SREBP-1 in MHCC97-H cells enhanced the cytotoxic effect of Sorafenib; the IC_50_ value decreased from 0.57 ± 0.07 to 0.12 ± 0.01 μmol/L (Fig. 3a). The resistance index (RI) was 4.97. On the other hand, overexpression of SREBP-1 in MHCC97-L cells resisted better to Sorafenib; the IC_50_ values increased from 0.78 ± 0.04 to 3.33 ± 0.23 μmol/L (Fig. 3b). Similarly, SREBP-1 knockdown synergized the effect of Sorafenib on cell metastasis (Supplementary Fig. 7). Moreover, Betulin administration augmented the killing effect of Sorafenib on MHCC97-H cells, with a decline of IC_50_ from 0.58 ± 0.06 to 0.15 ± 0.02 μmol/L (Fig. 3c), and facilitated Sorafenib-mediated suppression on metastasis (Fig. 3d). These in vitro data indicate that SREBP-1 induces Sorafenib resistance in HCC cells, suggesting the inhibition of SREBP-1 as a strategy to overcome chemoresistance.

### Betulin facilitates Sorafenib’s antitumor effect on s.c. HCC tumors

We next examined the effect of SREBP-1 inhibition on Sorafenib’s effects in vivo. We first found that Betulin oral gavage administration significantly suppressed s.c. MHCC97-H tumor growth (Supplementary Fig. 8). Gene expression analysis showed that Betulin treatment decreased glucose uptake, LDH activity, and lactate and ATP production in tumor cells in a dose-dependent manner (Supplementary Fig. 9A, B). Furthermore, gene expression analysis of isolated tumor cells showed that Betulin treatment reduced fatty acid and glucose metabolisms and EMT in tumors (Supplementary Fig. 9C and Supplementary Table 6). In order to test whether Betulin works synergistically with Sorafenib on HCC tumors, we administered Betulin at 2 mg/kg, the mild concentration which did not have an obvious tumor rejection effect but had SREBP-1 inhibition activity, in the following experiments.

**Fig. 4:**
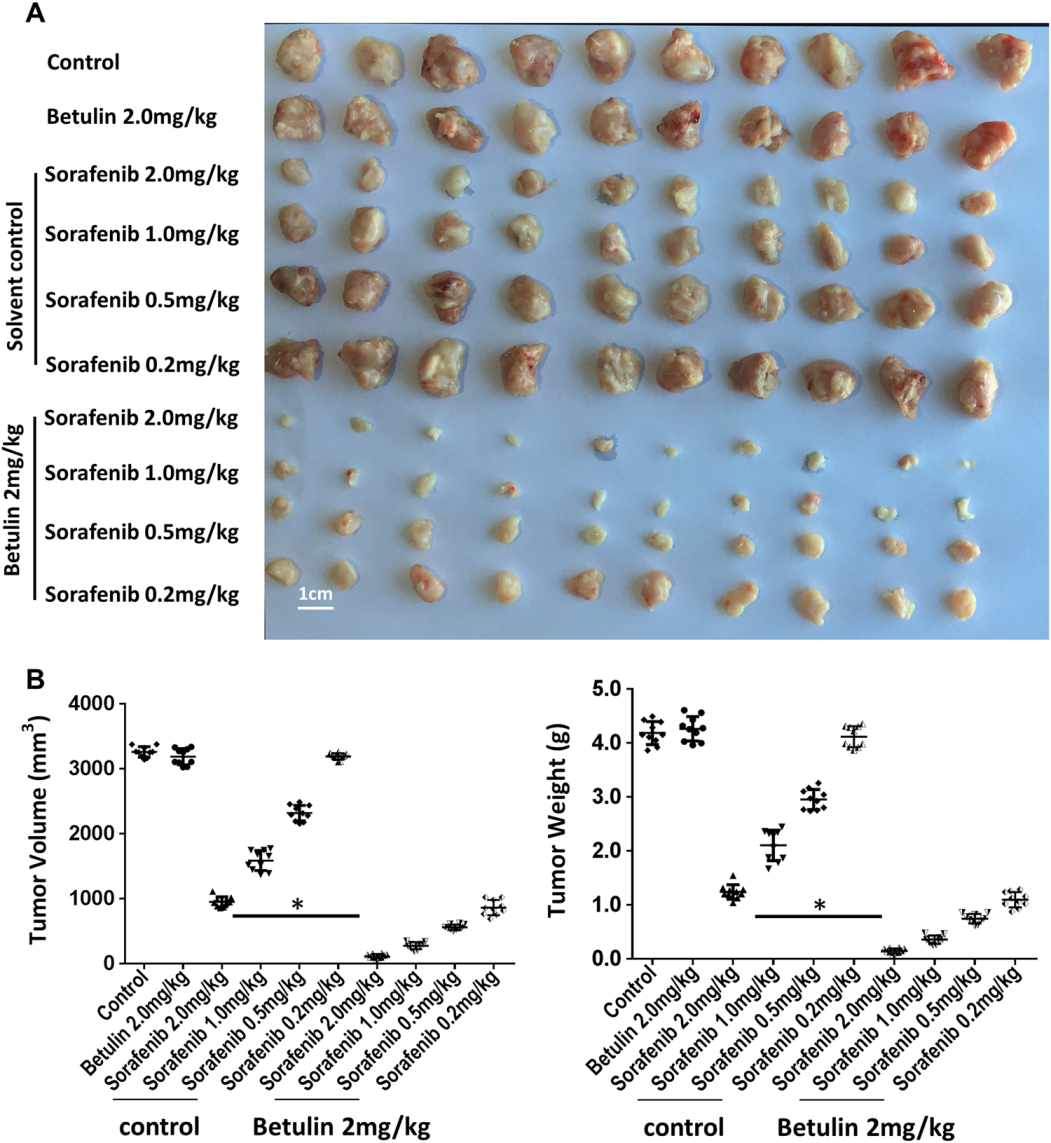
Betulin synergizes Sorafenib’s effect on HCC s.c. tumor growth. **a** MHCC97-H cells were injected into nude mice subcutaneously. At day 6, mice started receiving vehicle control, or the indicated concentrations of Sorafenib, or 2 mg/kg Betulin or the indicated concentration of Sorafenib + 2 mg/kg Betulin orally every other day 10 times. At day 21 post treatment, mice were killed and tumors were obtained (*N* = 10). **b** Quantitative results of tumor volume and tumor weight from **a**

**Fig. 5:**
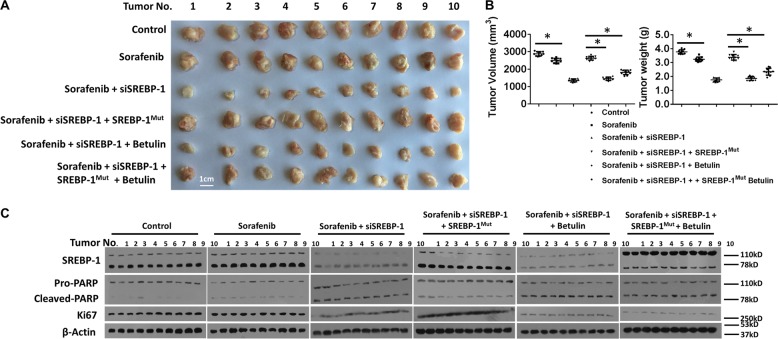
SREBP-1 knockdown synergizes Sorafenib’s effect on HCC s.c. tumor growth. **a** MHCC97-H s.c. tumors were established by using MHCC97-H cells transfected with control or SREBP-1 siRNAs, or SREBP-1 siRNAs plus SREBP-1^mut^ expressing vector. MHCC97-H s.c. tumor-bearing mice received vehicle control, or Sorafenib at the IC_50_ concentration, or 2 mg/kg Betulin or Sorafenib + Betulin orally every other day 10 times. At day 21 post treatment, mice were killed and tumors were obtained (*N* = 10). **b** Quantitative results of tumor volume and tumor weight from **a**. **c** Western blotting of SREBP-1 and apoptosis and proliferation-associated proteins in tumors from **a**. β-actin was used as internal control

When combined with Sorafenib, Betulin significantly enhanced the growth of MHCC97-H cells in s.c. tumors, compared with Sorafenib treatment (Fig. 4). This result suggests that Betulin and Sorafenib synergistically reject HCC tumors. We next examined the specificity of Betulin on promoting Sorafenib’s antitumor effect in vivo. We administered HCC s.c. tumors with SREBP-1 siRNA during Sorafenib or Sorafenib and Betulin combination treatments, and found that Betulin could not further control the growth of SREBP-1 knockdown tumors (Fig. 5a, b). However, the effect induced by SREBP-1 knockdown was rescued by ectopic expression of a mutated form of SREBP-1 (SREBP-1^mut^), which cannot be targeted by the siRNA (Fig. 5a, b). On the other hand, SREBP-1-overexpressed HCC tumors resisted to Sorafenib treatment, but were susceptible to Sorafenib and Betulin combinations (Supplementary Fig. 10A, B). These observations suggest that Betulin facilitates Sorafenib’s effect through SREBP-1 inhibition specifically. Sorafenib induces tumor regression through tumor cell apoptosis. Thus, we tested the level of pro-PARP, cleaved-PARP, and Ki67 in s.c. tumors treated as described above. We found that the knockdown of SREBP-1 promoted apoptosis and restricted cell proliferation, whereas SREBP-1 overexpression reduced cell apoptosis (Fig. 5c and Supplementary Fig. 10C).

### Betulin improves Sorafenib-mediated blockade of HCC tumor in situ growth

**Fig. 6:**
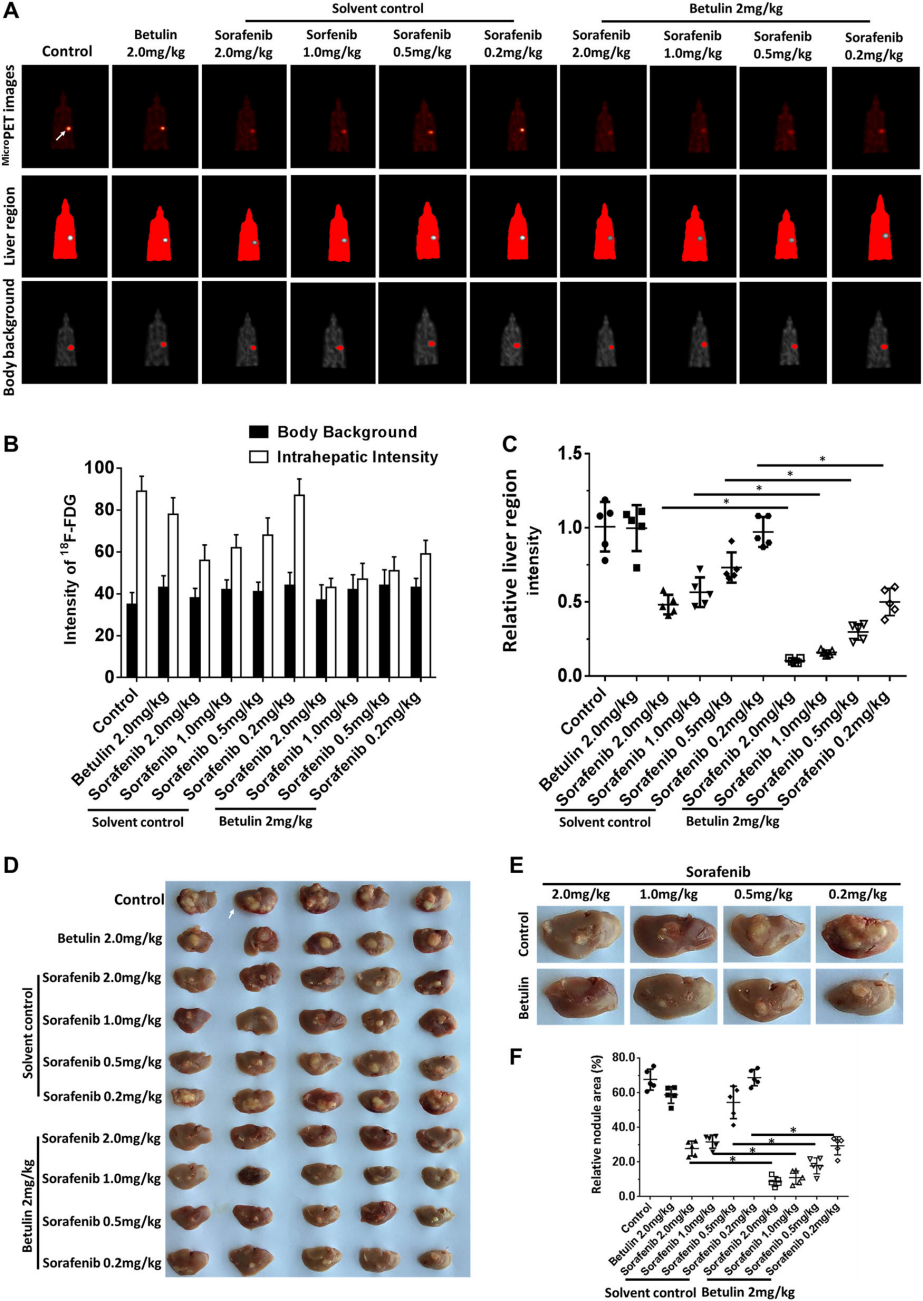
Betulin synergizes Sorafenib’s effect on HCC in situ growth in the mouse liver. **a** The in situ xenograft tumors were established by injected MHCC97-H cells directly into mouse livers. Tumor-bearing mice then received vehicle control, or the indicated concentrations of Sorafenib, or 2 mg/kg Betulin or the indicated concentration of Sorafenib + 2 mg/kg Betulin orally every other day 10 times. At day 21 post treatment, mice were scanned by in vivo small-animal MicroPET imaging (*N* = 10). The images show the in situ tumors. **b** The ^18^F-FDG intensity analysis and quantification results. **c** Relative inhibitory rate of tumor growth calculated from (b). **d** Livers from control or tumor-bearing mice from **a**. **e** Representative tumor nodules from each treatment group. **f** Quantification results from **e**

To study the effect of Betulin on in situ HCC tumor growth during Sorafenib treatment, we established in situ HCC tumors in nude mice livers by using MHCC97-H cell implantation. We then performed Sorafenib or Sorafenib plus Betulin treatment every other day 10 times in total and in the endpoint (day 21 post injection) examined tumor burden by PET/CT scanning. The analysis of the imaging results indicated that Betulin enhanced the effect of Sorafenib upon blocking HCC in situ growth (Fig. 6a–c). Meanwhile, we collected liver tissues and measured tumor lesions (Fig. 6d). Our data showed that the tumor nodule areas of Betulin- and Sorafenib-treated mice were smaller than those of Sorafenib-treated or control mice (Fig. 6e, f). These results indicate that Betulin synergizes Sorafenib in blocking HCC in situ growth, which supports our findings in HCC s.c. tumor treatments. To further study the specificity of Betulin, we performed Sorafenib or Sorafenib plus Betulin treatment upon SREBP-1 knockdown or SREBP-1 overexpressing HCC in situ tumor-bearing mice. We found that SREBP-1 knockdown diminished the effect of Betulin upon synergizing Sorafenib (Supplementary Fig. 11), whereas Betulin exerted a considerable synergistic effect upon treating SREBP-1-overexpressing tumors (Supplementary Fig. 12). These results suggested that Betulin synergizes Sorafenib’s effect upon controlling in situ HCC tumors by targeting SREBP-1.

## Discussion

Tumors are characterized by intensive anaerobic glycolysis, which benefits tumor cells via providing quick energy for cell proliferation, altering the hostile tumor microenvironment for immune cell infiltration, and inducing drug resistance^11,53,54^. Therefore, targeting glucose metabolism-related pathways is considered as an effective approach to control tumor growth and enhance the efficacy of antitumor chemotherapy. SREBP-1 is one of the major regulators of cellular lipid metabolism^25–27^; it controls lipid synthesis via transcriptional regulation of its downstream genes: FASN, ACC, ACLY, SCD, and so on^25–27^. Due to the close correlation of glucose and lipid metabolisms, SREBP-1 is also known to regulate glucose and glutamine metabolic pathways^25–27,54^. Betulin can inhibit SREBP family protein activation by directly binding to the SCAP region of this molecule which further blocks the cleavage of SREBP. Li et al. have reported that inhibiting SREBPs by Betulin can suppress HCC tumor-associated inflammation^55^. Inflammation in tumors, specifically induced by chronic viral infection, is critical for tumor-associated immune suppression and drug resistance^56–59^. Thus, we hypothesized that Betulin treatment on HCC tumor models can induce tumor regression and reduce drug resistance against chemotherapy. In the present study, we showed that a high level of SREBP-1 correlates with poor prognosis of HCC patients treated with the chemotherapy agent Sorafenib. Our study provides evidence that SREBP-1 protects the tumor cell via enhancing glycolytic activities and SREBP-1 inhibition induces cell death and promotes the antitumor effect of Sorafenib. These results indicated that SREBP-1 is critical for HCC tumor development and targeting to SREBP-1 is therapeutically effective for HCC treatment.

The Warburg effect is characterized by the transformation of the energy acquisition from oxidative phosphorylation to anaerobic glycolysis, which is regarded as a hallmark of tumor cells. Intracellular glucose can be hydrolyzed into pyruvate during glycolysis; then pyruvate goes into the tricarboxylic acid cycle when the oxygen supply is sufficient and energy demand is low^11,60^. However, in the hypoxic tumor microenvironment, where energy demand is very high, pyruvate is metabolized into lactate acid via anaerobic glycolysis, and glucose uptake is enhanced to increase the ATP production in tumor cells^61–63^. To block glycolytic activity during HCC treatment, there are three strategies: (1) using inhibitors of glucose transporters^63,64^, (2) using inhibitors targeting hypoxia-associated pathways^11,65–67^, and (3) inhibitors of LDHA^68^ to ameliorate the accumulation of lactic acid. Hypersynthesis of fatty acid in HCC cells is driving glucose uptake^61–65^. Inhibition of SREBP-1 represses lipid metabolism in tumor cells, and in turn inhibits glucose uptake and glycolysis^69–71^. In this study, we showed that knockdown or inhibition of SREBP-1 in HCC cells impaired glucose uptake, LDH activity, and reduced ATP and lactate production, which provide mechanistic insights into the SREBP-1 function.

As a primary therapeutic choice of HCC treatment, Sorafenib does not deliver a robust effect due to the subsequent resistance that occurs soon after the initial treatment^9^. It has been reported that inhibition of lipid metabolism effectively represses the growth and metastasis of tumor cells and reduces Sorafenib resistance^72–74^. Thus, the inhibition of cellular metabolisms which facilitate tumor cell survival may enhance the efficacy of therapeutic chemodrugs. Our results indicated that Betulin as the SREBP-1 inhibitor overcomes the resistance of HCC cells to Sorafenib. Further studies will attempt to address two aspects: first, study the mechanism by which Betulin-treated cells or SREBP-1 knockdown cells become less resistant to Sorafenib; second, prove the accessibility that SREBP-1 becomes a prognosis marker of HCC patients.

## Supplementary information

Supplementary Figure 1

Supplementary Figure 2

Supplementary Figure 3

Supplementary Figure 4

Supplementary Figure 5

Supplementary Figure 6

Supplementary Figure 7

Supplementary Figure 8

Supplementary Figure 9

Supplementary Figure 10

Supplementary Figure 11

Supplementary Figure 12

Supplementary Table 1

Supplementary Table 2

Supplementary Table 3

Supplementary Table 4

Supplementary Table 5

Supplementary Table 6

supplementary figure legends
