# Supplementary figures and images for: SREBP-1 inhibitor Betulin enhances the antitumor effect of Sorafenib on hepatocellular carcinoma via restricting cellular glycolytic activity
Source: Cell Death Dis. 2019 Sep 11;10(9):672. doi: 10.1038/s41419-019-1884-7 (PMC6739379; doi:10.1038/s41419-019-1884-7)

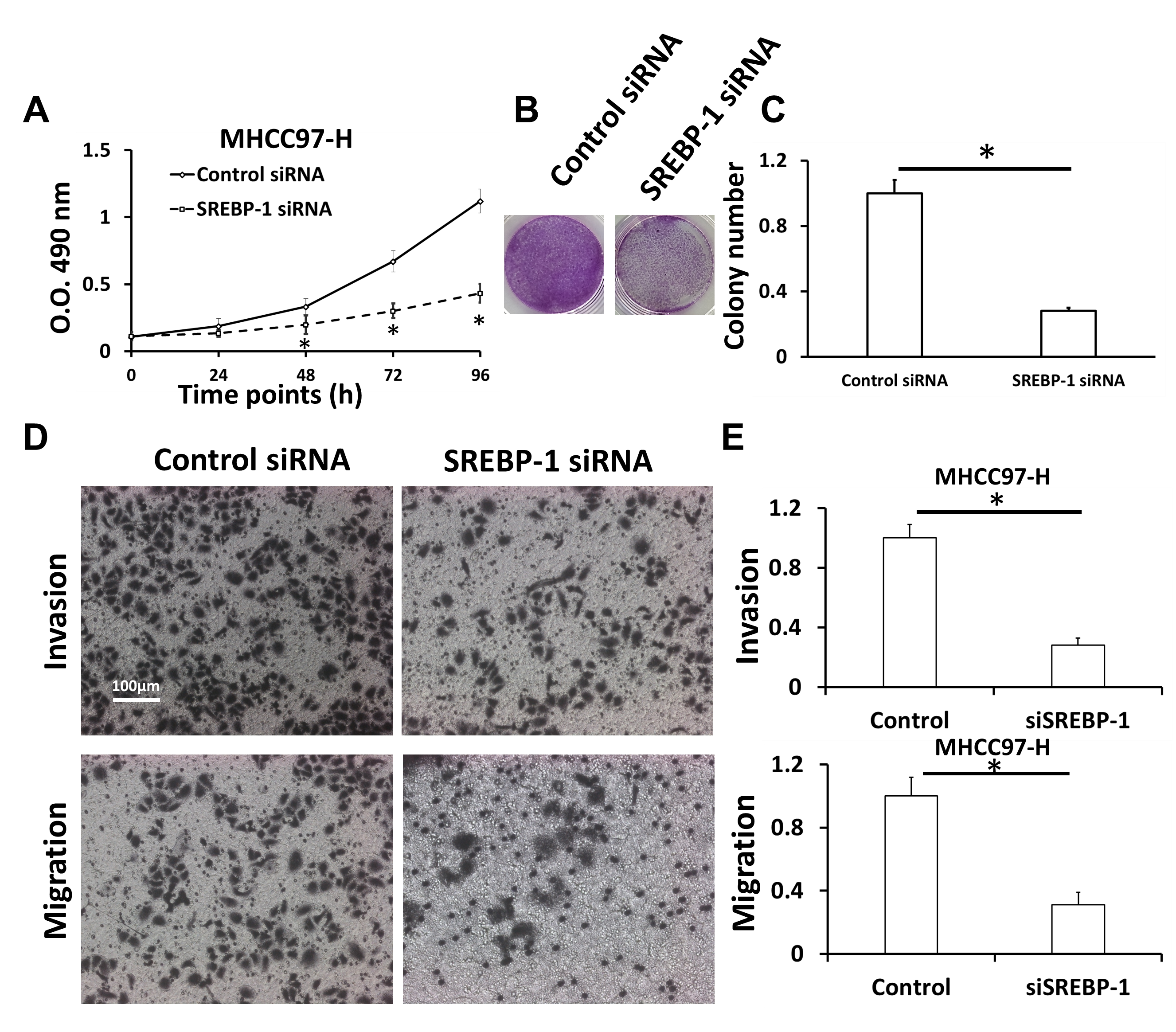

Supplement: Supplementary file 1 — Supplementary Figure 1 [file 41419_2019_1884_MOESM1_ESM.tif]

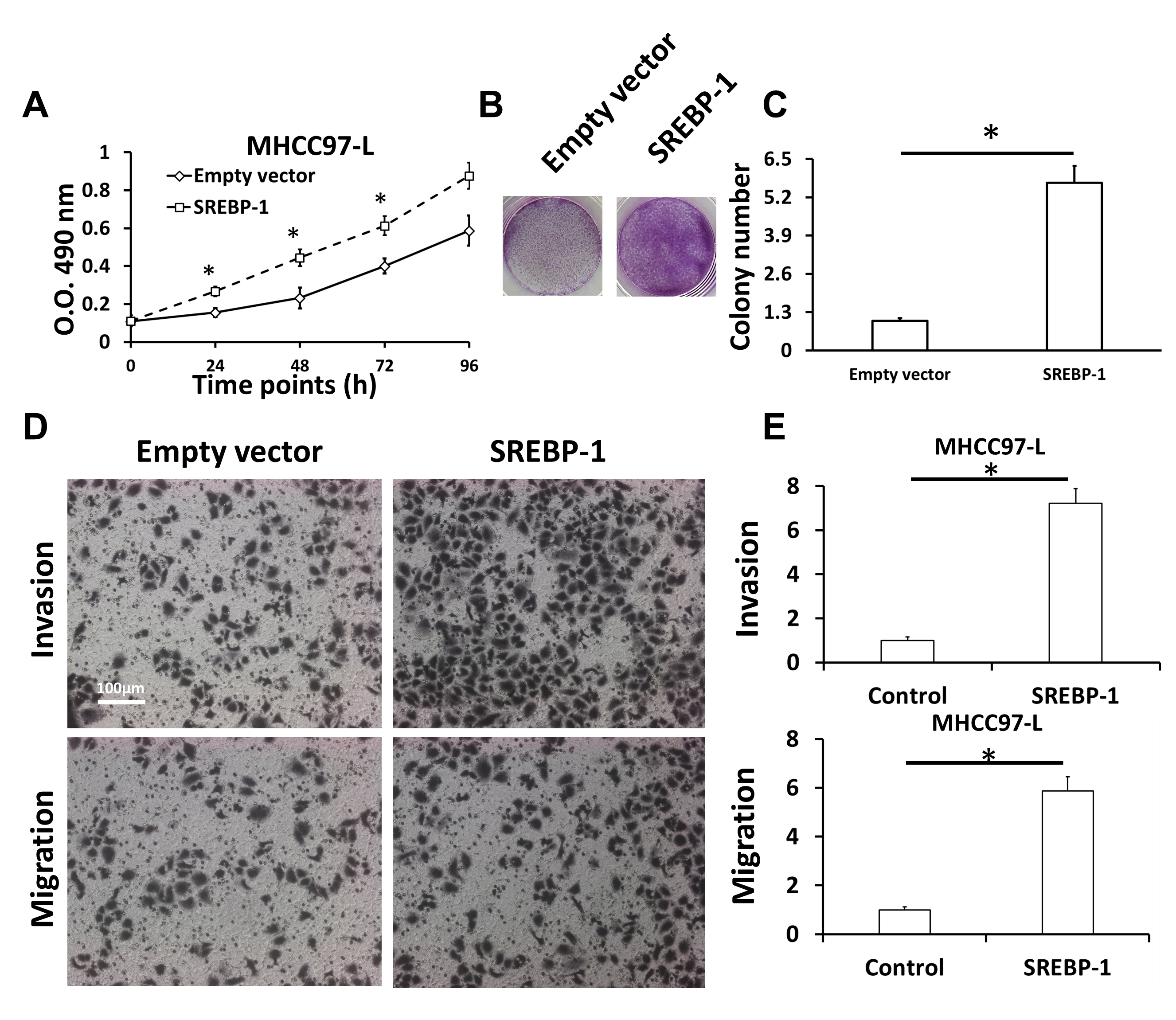

Supplement: Supplementary file 2 — Supplementary Figure 2 [file 41419_2019_1884_MOESM2_ESM.tif]

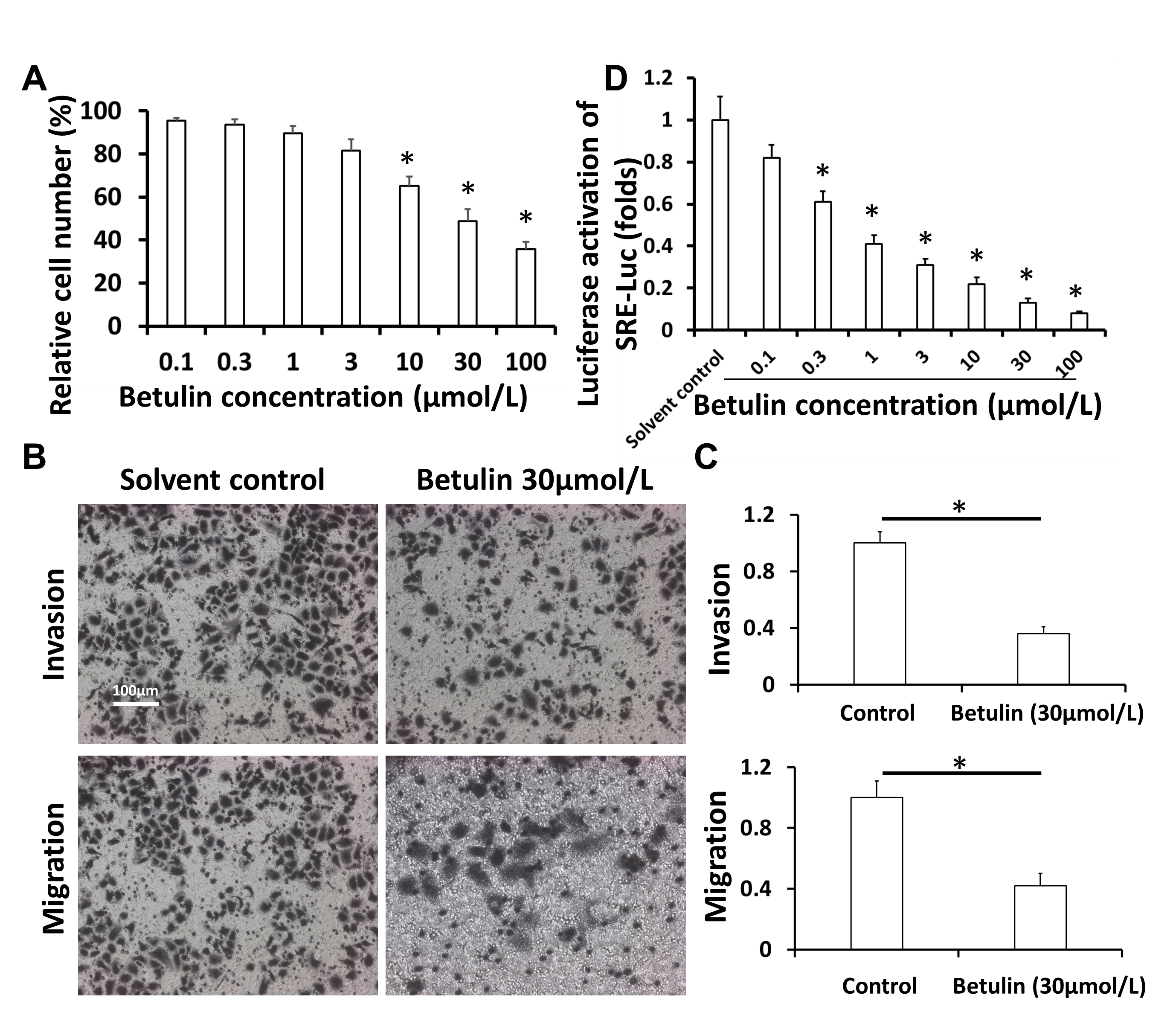

Supplement: Supplementary file 3 — Supplementary Figure 3 [file 41419_2019_1884_MOESM3_ESM.tif]

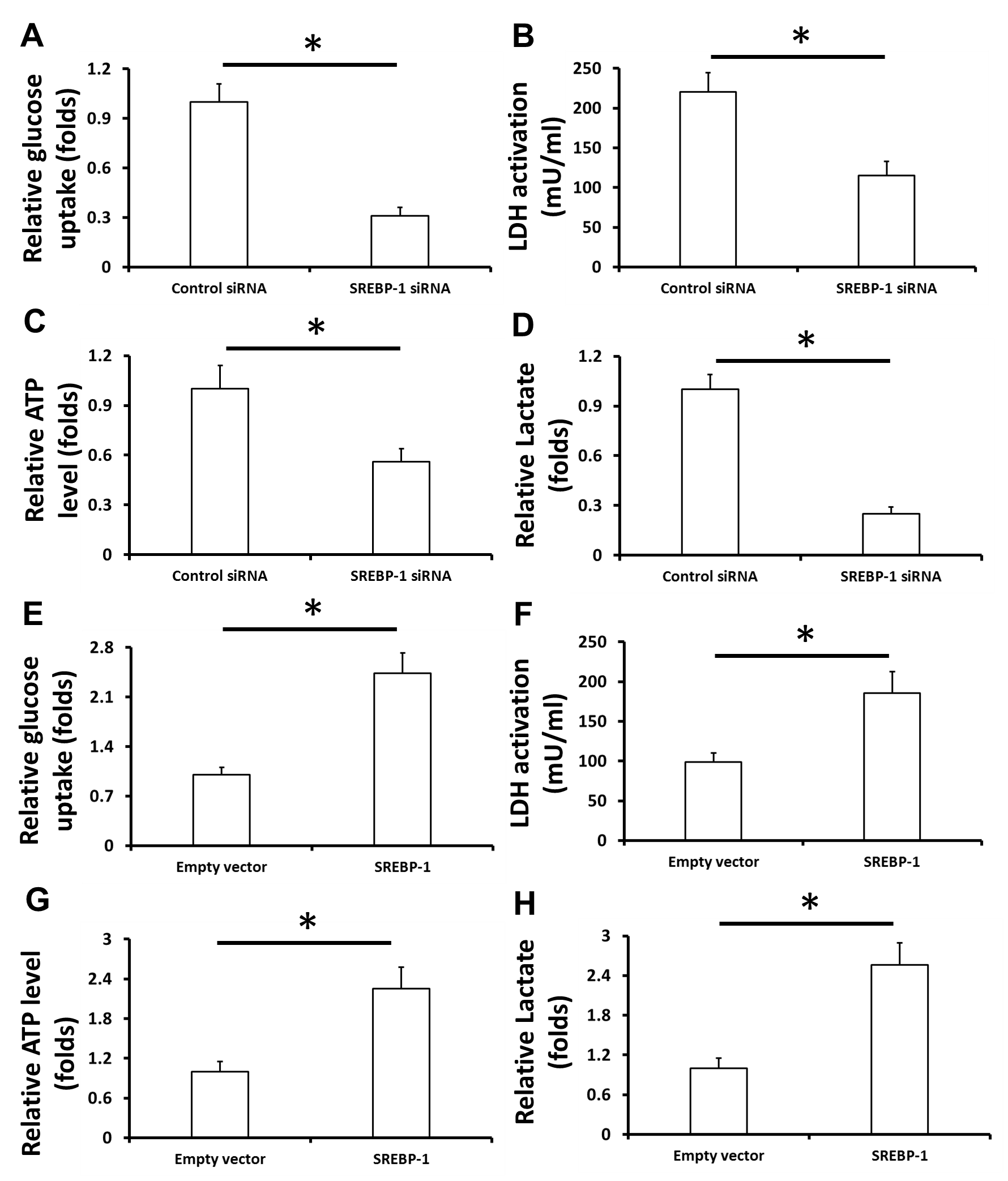

Supplement: Supplementary file 4 — Supplementary Figure 4 [file 41419_2019_1884_MOESM4_ESM.tif]

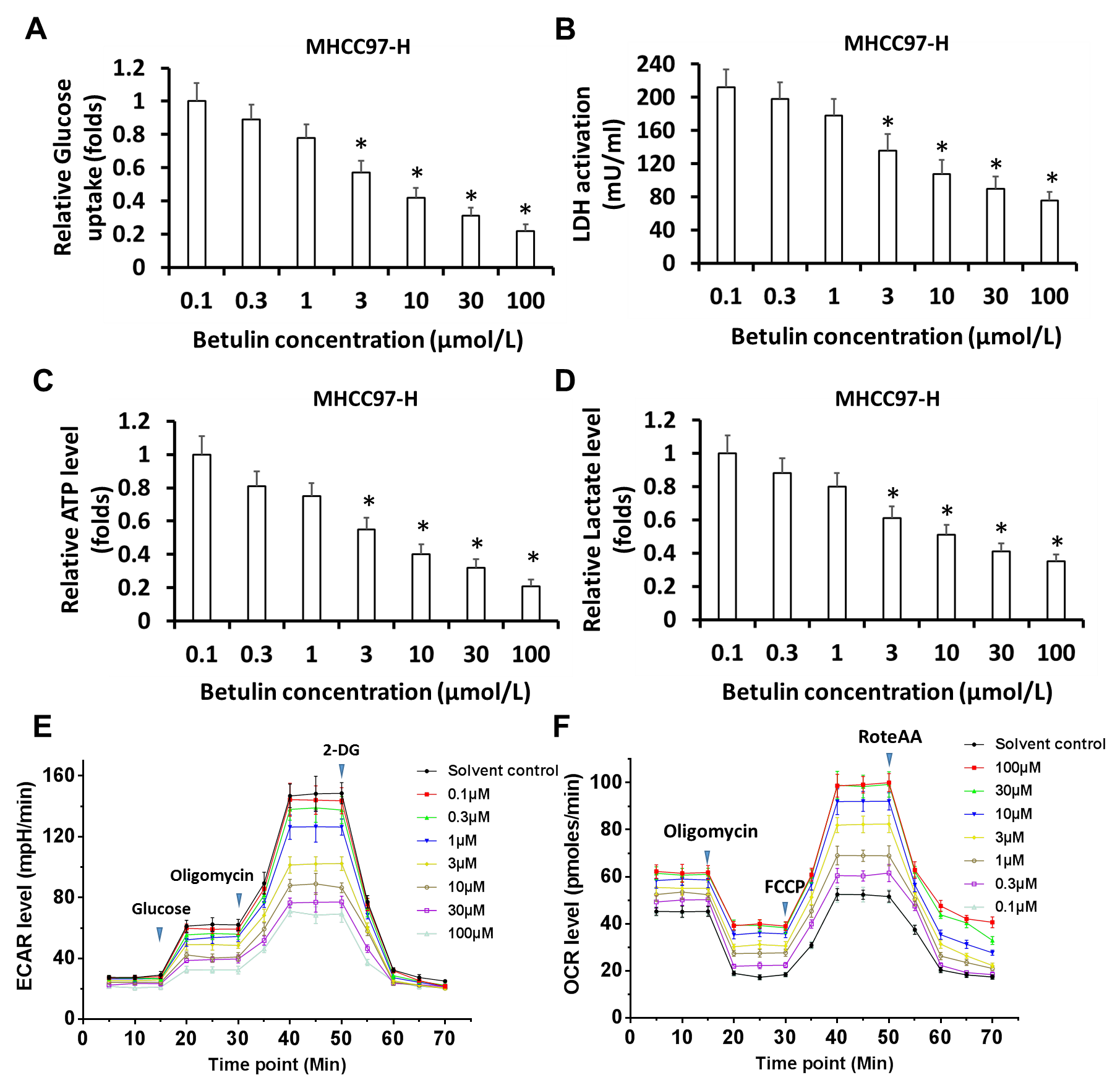

Supplement: Supplementary file 5 — Supplementary Figure 5 [file 41419_2019_1884_MOESM5_ESM.tif]

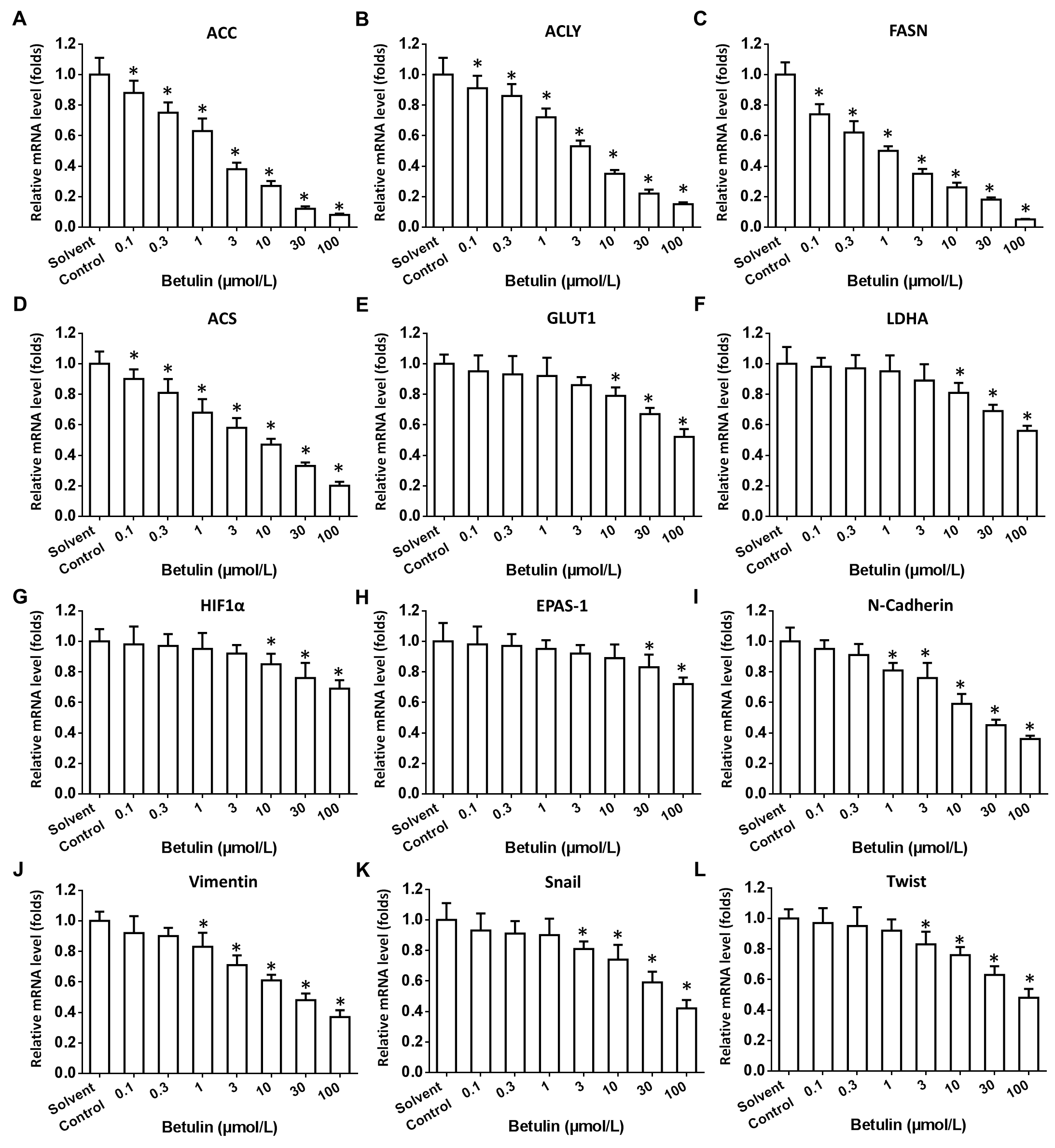

Supplement: Supplementary file 6 — Supplementary Figure 6 [file 41419_2019_1884_MOESM6_ESM.tif]

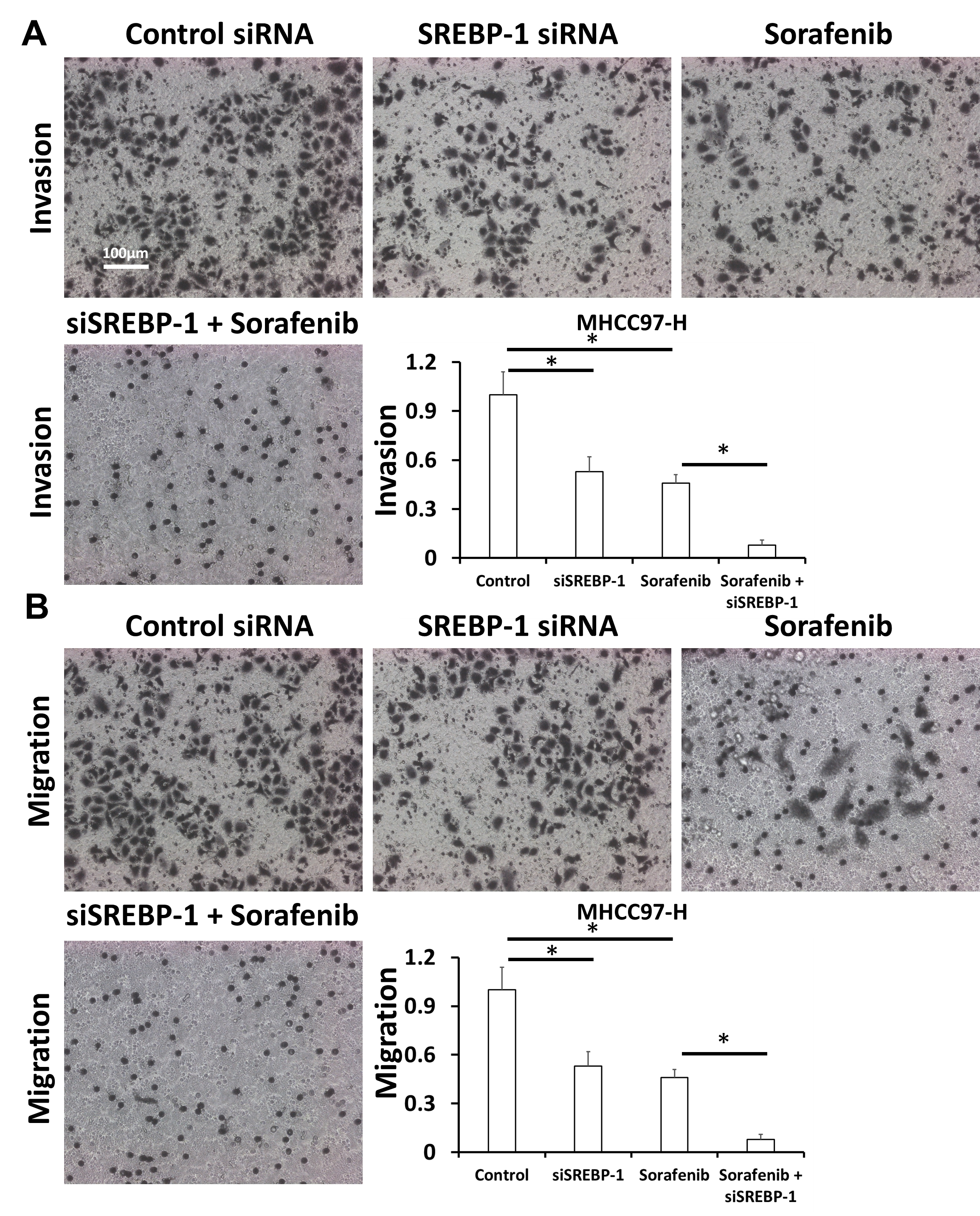

Supplement: Supplementary file 7 — Supplementary Figure 7 [file 41419_2019_1884_MOESM7_ESM.tif]

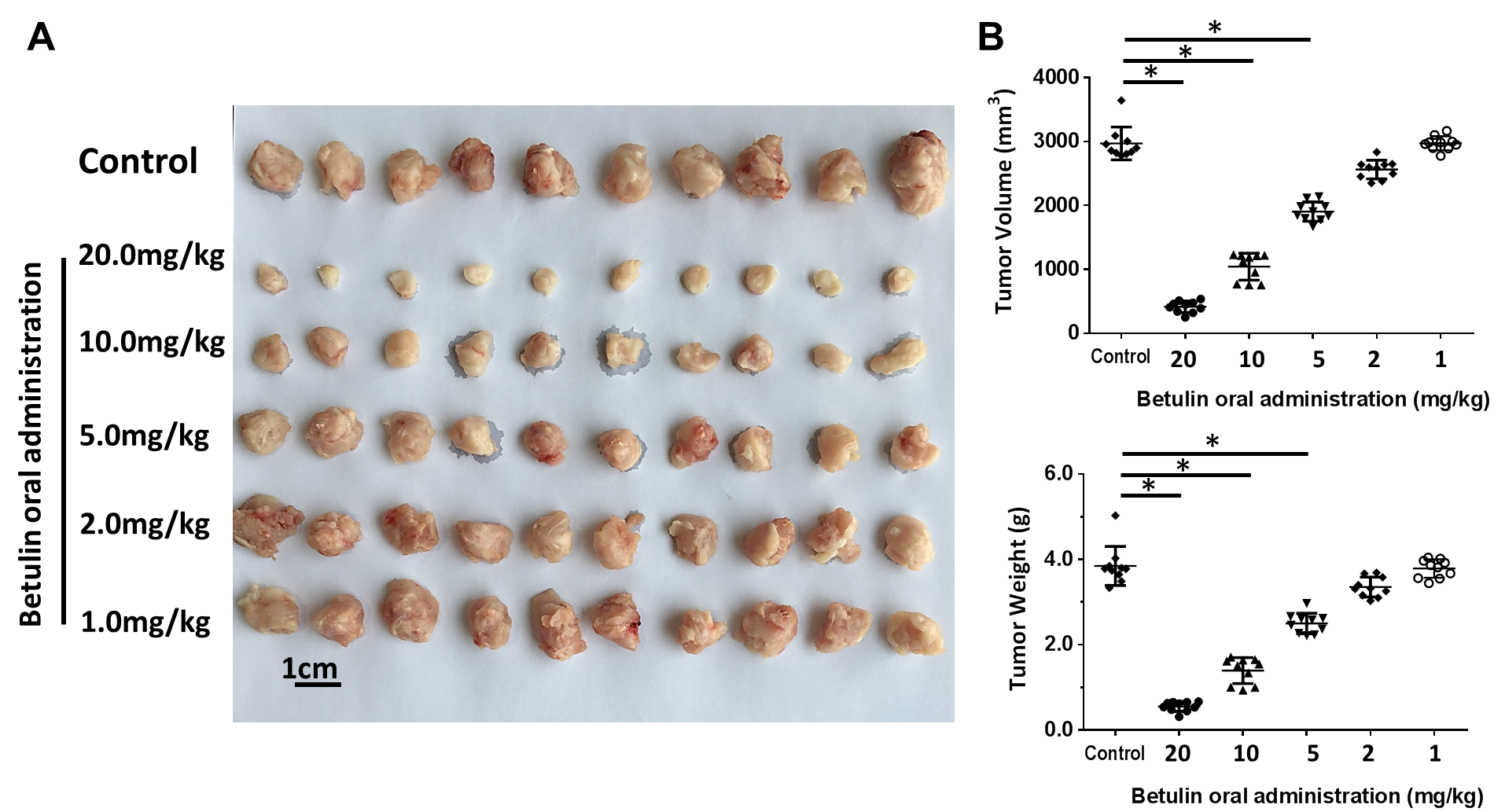

Supplement: Supplementary file 8 — Supplementary Figure 8 [file 41419_2019_1884_MOESM8_ESM.tif]

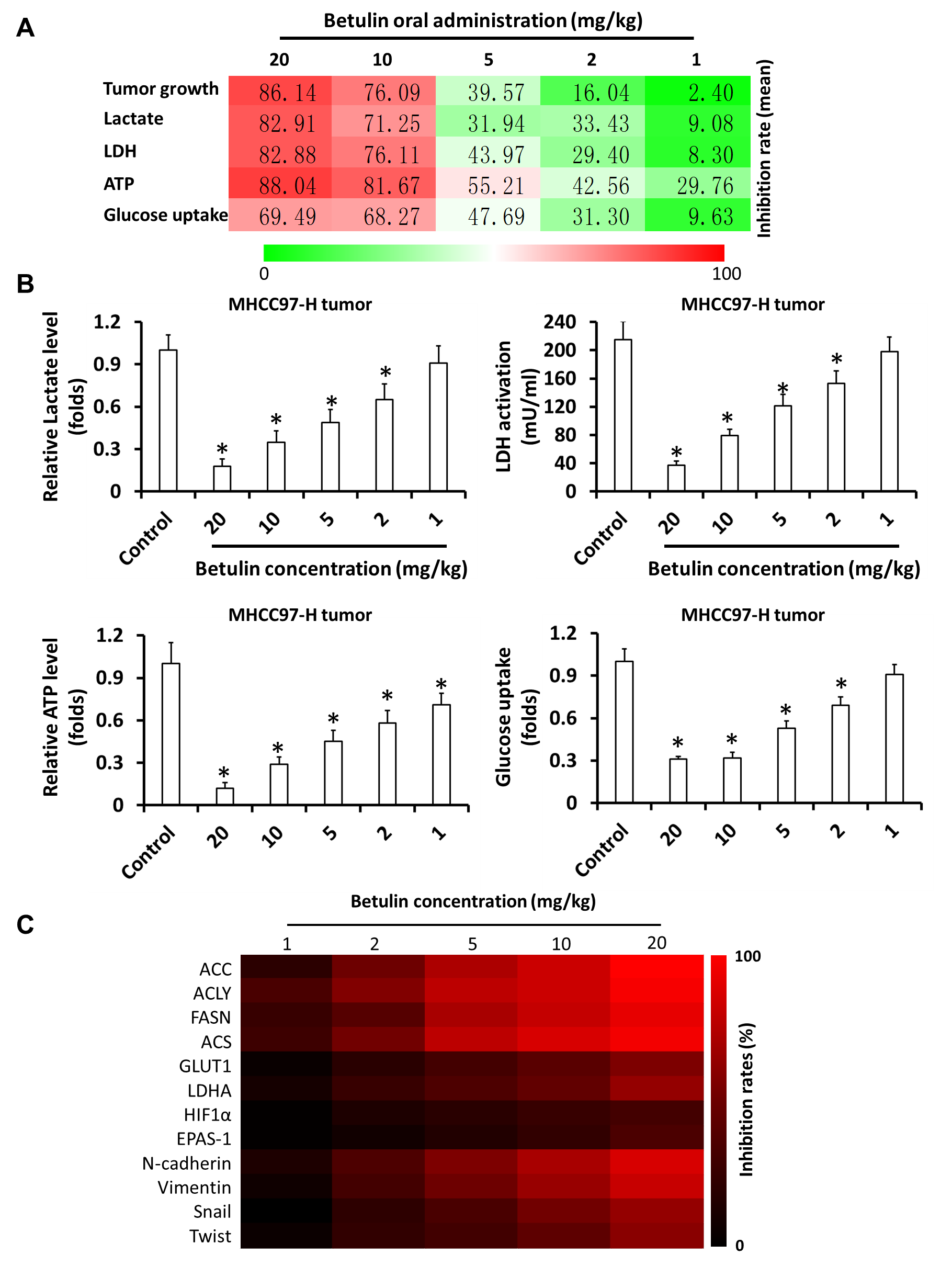

Supplement: Supplementary file 9 — Supplementary Figure 9 [file 41419_2019_1884_MOESM9_ESM.tif]

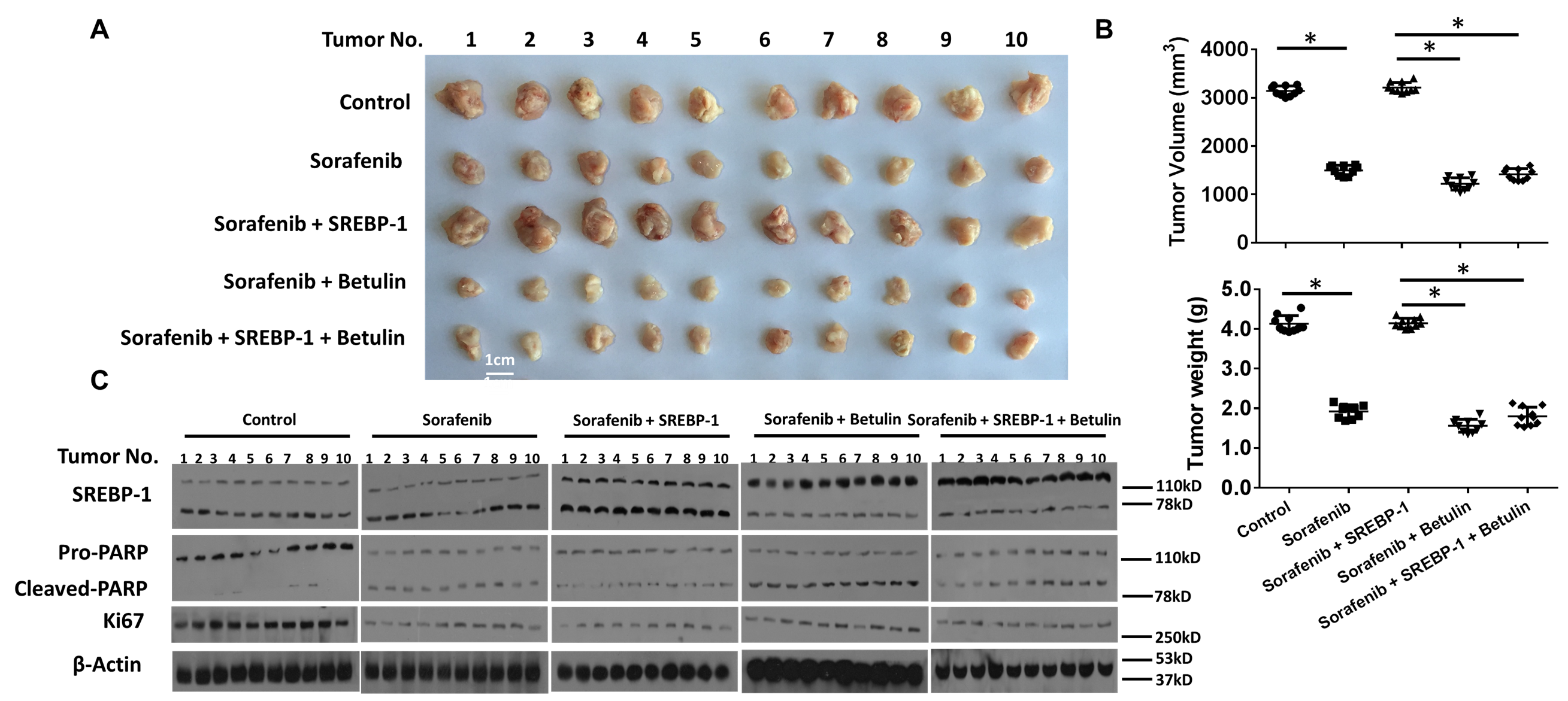

Supplement: Supplementary file 10 — Supplementary Figure 10 [file 41419_2019_1884_MOESM10_ESM.tif]

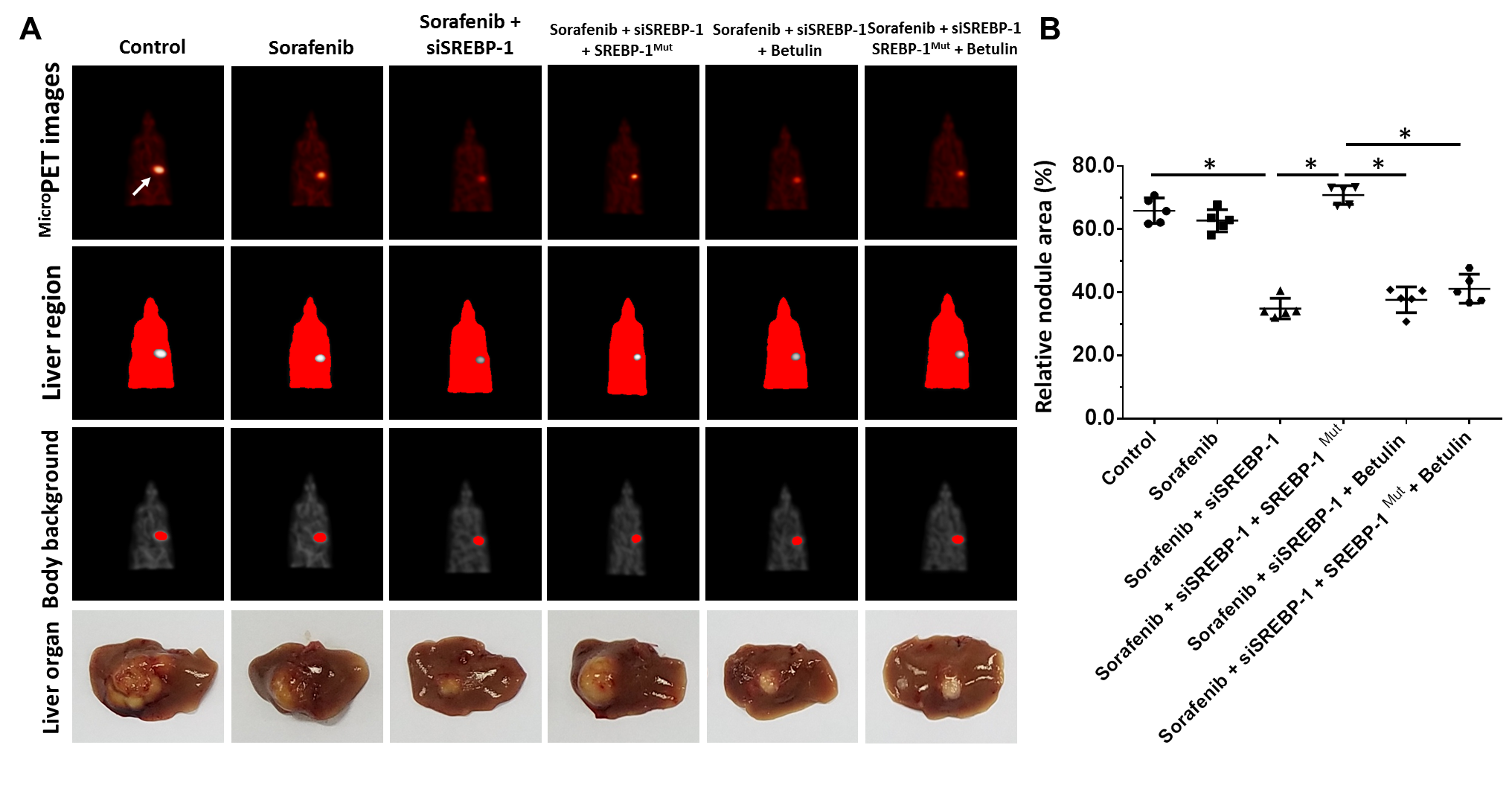

Supplement: Supplementary file 11 — Supplementary Figure 11 [file 41419_2019_1884_MOESM11_ESM.tif]

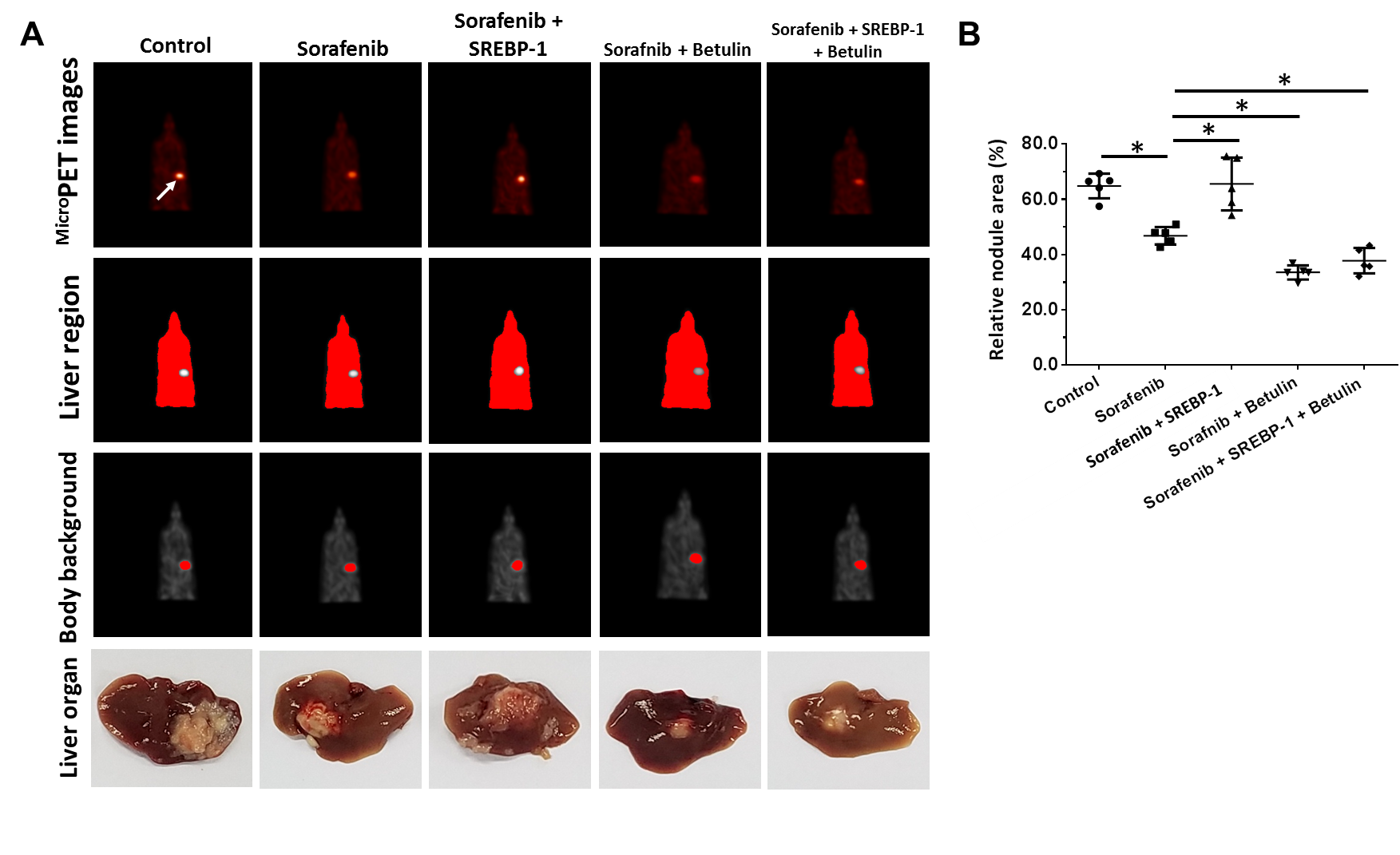

Supplement: Supplementary file 12 — Supplementary Figure 12 [file 41419_2019_1884_MOESM12_ESM.tif]
