# Supplementary material for: SREBP-1 inhibitor Betulin enhances the antitumor effect of Sorafenib on hepatocellular carcinoma via restricting cellular glycolytic activity
Source: Cell Death Dis. 2019 Sep 11;10(9):672. doi: 10.1038/s41419-019-1884-7 (PMC6739379; doi:10.1038/s41419-019-1884-7)
Supplement: Supplementary file 13 — Supplementary Table 1 [file 41419_2019_1884_MOESM13_ESM.docx]

**Supplemental tables:**

**Table 1. Baseline clinical data of 52 patients with advanced HCC.**

| **Presentation** | **Case** (%) |
| --- | --- |
| Median age, yr (range) | 51 (24-74) |
| Gender, male (%) | 47 (90.38%) |
| Aetiology (%) |  |
| HBV positive | 44 (84.61%) |
| HCV positive | 8 (15.39%) |
| ECOG PS (%) |  |
| 0 | 16 (30.76%) |
| 1 | 28 (53.84%) |
| 2 | 6 (15.40%) |
| AFP (%) |  |
| Normal | 12 (23.10%) |
| Elevated | 40 (76.90%) |
| extrahepatic metastasis (%) | 34 (65.38%) |
| LN metastasis (%) | 23 (44.23%) |
| Portal vein invasion (%) | 36 (69.23%) |
| Chilg-Pugh (%) |  |
| A | 8 (15.38%) |
| B | 32 (61.53%) |
| C | 12 (23.09%) |
| Median size of index tumor, cm (range) | 4.2 (1-13.3) |
| Median number of index tumors | 3 (1-5) |
| Prior local therapy (%) | None |
