# Supplementary material for: SREBP-1 inhibitor Betulin enhances the antitumor effect of Sorafenib on hepatocellular carcinoma via restricting cellular glycolytic activity
Source: Cell Death Dis. 2019 Sep 11;10(9):672. doi: 10.1038/s41419-019-1884-7 (PMC6739379; doi:10.1038/s41419-019-1884-7)
Supplement: Supplementary file 14 — Supplementary Table 2 [file 41419_2019_1884_MOESM14_ESM.docx]

**Supplemental table 2 Concentrations (μmol/L) Butulin or Sorafenib used in cell based experiments**

| **Agents** | **Butulin** | **Sorafenib** |
| --- | --- | --- |
| **Concentrations (μmol/L)** | 100 | 10 |
|  | 30 | 3 |
|  | 10 | 1 |
|  | 3 | 0.3 |
|  | 1 | 0.1 |
|  | 0.3 | 0.03 |
|  | 0.1 | 0.01 |
