# Supplementary material for: SREBP-1 inhibitor Betulin enhances the antitumor effect of Sorafenib on hepatocellular carcinoma via restricting cellular glycolytic activity
Source: Cell Death Dis. 2019 Sep 11;10(9):672. doi: 10.1038/s41419-019-1884-7 (PMC6739379; doi:10.1038/s41419-019-1884-7)
Supplement: Supplementary file 15 — Supplementary Table 3 [file 41419_2019_1884_MOESM15_ESM.docx]

**Supplemental Table 3 Primers used in this work**

| **Targets** | **Primers** | **Sequences** |
| --- | --- | --- |
| **SREBP-1** | Forward Sequence | ACTTCTGGAGGCATCGCAAGCA |
|  | Reverse Sequence | AGGTTCCAGAGGAGGCTACAAG |
| **ACC** | Forward Sequence | TTCACTCCACCTTGTCAGCGGA |
|  | Reverse Sequence | GTCAGAGAAGCAGCCCATCACT |
| **ACLY** | Forward Sequence | GCTCTGCCTATGACAGCACCAT |
|  | Reverse Sequence | GTCCGATGATGGTCACTCCCTT |
| **FASN** | Forward Sequence | TTCTACGGCTCCACGCTCTTCC |
|  | Reverse Sequence | GAAGAGTCTTCGTCAGCCAGGA |
| **ACS** | Forward Sequence | ATCAGGCTGCTCATGGATGACC |
|  | Reverse Sequence | AGTCCAAGAGCCATCGCTTCAG |
| **GLUT1** | Forward Sequence | TTGCAGGCTTCTCCAACTGGAC |
|  | Reverse Sequence | CAGAACCAGGAGCACAGTGAAG |
| **LDHA** | Forward Sequence | GGATCTCCAACATGGCAGCCTT |
|  | Reverse Sequence | AGACGGCTTTCTCCCTCTTGCT |
| **HIF1α** | Forward Sequence | TATGAGCCAGAAGAACTTTTAGGC |
|  | Reverse Sequence | CACCTCTTTTGGCAAGCATCCTG |
| **EPAS-1** | Forward Sequence | CTGTGTCTGAGAAGAGTAACTTCC |
|  | Reverse Sequence | TTGCCATAGGCTGAGGACTCCT |
| **N-cadherin** | Forward Sequence | CCTCCAGAGTTTACTGCCATGAC |
|  | Reverse Sequence | GTAGGATCTCCGCCACTGATTC |
| **Vimentin** | Forward Sequence | AGGCAAAGCAGGAGTCCACTGA |
|  | Reverse Sequence | ATCTGGCGTTCCAGGGACTCAT |
| **Snail** | Forward Sequence | TGCCCTCAAGATGCACATCCGA |
|  | Reverse Sequence | GGGACAGGAGAAGGGCTTCTC |
| **Twist** | Forward Sequence | GCCAGGTACATCGACTTCCTCT |
|  | Reverse Sequence | TCCATCCTCCAGACCGAGAAGG |
