# Supplementary material for: SREBP-1 inhibitor Betulin enhances the antitumor effect of Sorafenib on hepatocellular carcinoma via restricting cellular glycolytic activity
Source: Cell Death Dis. 2019 Sep 11;10(9):672. doi: 10.1038/s41419-019-1884-7 (PMC6739379; doi:10.1038/s41419-019-1884-7)
Supplement: Supplementary file 16 — Supplementary Table 4 [file 41419_2019_1884_MOESM16_ESM.docx]

**Supplemental Table 4 The concentrations (mg/kg) of Betulin or Sorafenib used in animal experiments.**

| **Agents** | **Betulin** | **Sorafenib** |
| --- | --- | --- |
| **Concentrations (mg/kg)** | 20 | 2 |
|  | 10 | 1 |
|  | 5 | 0.5 |
|  | 2 | 0.2 |
|  | 1 |  |
