# Supplementary material for: SREBP-1 inhibitor Betulin enhances the antitumor effect of Sorafenib on hepatocellular carcinoma via restricting cellular glycolytic activity
Source: Cell Death Dis. 2019 Sep 11;10(9):672. doi: 10.1038/s41419-019-1884-7 (PMC6739379; doi:10.1038/s41419-019-1884-7)
Supplement: Supplementary file 17 — Supplementary Table 5 [file 41419_2019_1884_MOESM17_ESM.docx]

**Supplemental Table 5 the *IC_50_* values of Betulin on genes’ mRNA level from cell based experiments**

| Targets | *IC_50_* values of Betulin (μmol/L) |
| --- | --- |
| ACC | 1.87±0.15 |
| ACLY | 4.22±0.28 |
| FASN | 0.93±0.09 |
| ACS | 6.51±0.53 |
| GLUT1 | ~100 |
| LDHA | - |
| HIF1α | - |
| EPAS-1 | - |
| N-cadherin | 24.82±2.67 |
| Vimentin | 27.78±1.89 |
| Snail | 61.43±8.18 |
| Twist | 86.80±5.09 |

*IC_50_* values: half inhibitory effect concentration of agents
