# Supplementary material for: SREBP-1 inhibitor Betulin enhances the antitumor effect of Sorafenib on hepatocellular carcinoma via restricting cellular glycolytic activity
Source: Cell Death Dis. 2019 Sep 11;10(9):672. doi: 10.1038/s41419-019-1884-7 (PMC6739379; doi:10.1038/s41419-019-1884-7)
Supplement: Supplementary file 18 — Supplementary Table 6 [file 41419_2019_1884_MOESM18_ESM.docx]

**Supplemental Table 6 the *IC_50_* values of Betulin on genes’ mRNA level from animal experiments**

| Targets | *IC_50_* values of Betulin (mg/kg) |
| --- | --- |
| ACC | 1.77±0.19 |
| ACLY | 1.10±0.11 |
| FASN | 1.76±0.07 |
| ACS | 1.63±0.09 |
| GLUT1 | 7.66±0.16 |
| LDHA | 6.67±0.13 |
| HIF1α | - |
| EPAS-1 | ~20 |
| N-cadherin | 2.75±0.32 |
| Vimentin | 3.59±0.49 |
| Snail | 6.54±1.03 |
| Twist | 7.55±0.94 |

*IC_50_* values: half inhibitory effect concentration of agents
