# Supplementary material for: SREBP-1 inhibitor Betulin enhances the antitumor effect of Sorafenib on hepatocellular carcinoma via restricting cellular glycolytic activity
Source: Cell Death Dis. 2019 Sep 11;10(9):672. doi: 10.1038/s41419-019-1884-7 (PMC6739379; doi:10.1038/s41419-019-1884-7)
Supplement: Supplementary file 19 — supplementary figure legends [file 41419_2019_1884_MOESM19_ESM.docx]

**Supplemental Figure 1. SREBP-1 knock-down inhibits the proliferation and metastasis of HCC cells.** (A) MTT assay results from high metastatic MHCC97-H cells transfected with control or SREBP-1 siRNAs for 48 hours. (B) Representative results from colony formation assay using MHCC97-H cells transfected with control or SREBP-1 siRNAs for 48 hours. (C) Quantitative results from (B). (D-E) Transwell experiment using MHCC97-H cells transfected with control or SREBP-1 siRNAs for 48 hours: (D) representative images; (E) quantification results. All results were shown as mean±SD. Paired samples were tested by paired-sample *t*-test. Significant: * p<0.05 in all figures.

**Supplemental Figure 2 SREBP-1 overexpression enhances the proliferation and metastasis of HCC cells.** (A) MTT assay results from low metastatic MHCC97-L cells transfected with empty or SREBP-1 expressing vectors for 48 hours. (B) Representative results from colony formation assay using MHCC97-L cells transfected with empty or SREBP-1 expressing vectors for 48 hours. (C) Quantitative results from (B). (D-E) Transwell experiment using MHCC97-L cells transfected with empty or SREBP-1 expressing vectors for 48 hours: (D) representative images; (E) quantification results.

**Supplemental Figure 3 Betulin suppresses the proliferation and metastasis of HCC cells.** (A) MTT assay results from MHCC97-H cells treated with indicated concentrations of Betulin (100μmol/L, 30μmol/L, 10μmol/L, 3μmol/L, 1μmol/L, 0.3μmol/L or 0.1μmol/L) for 48 hours. (B-C) Transwell experiment using MHCC97-L cells treated with indicated concentrations of Betulin 48 hours: (B) representative images; (C) quantification results. (D) MHCC97-H cells were transfected with control vectors or SRE-Luc (SREBP-1 response element luciferase reporter) vectors, then treated with indicated concentrations of Betulin for 48 hours. Cells were harvested for luciferase experiments.

**Supplemental Figure 4. SREBP-1 regulates the glycolytic activities of HCC cells.** (A) The glucose uptake capacity, (B) LDH enzymatic activity, (C) ATP and (D) lactate productions of MHCC97-H cells transfected with control or SREBP-1 siRNAs for 48 hours. (E) The glucose uptake capacity, (F) LDH enzymatic activity, (G) ATP and (H) lactate productions of MHCC97-L cells transfected with empty or SREBP-1 expressing vectors for 48 hours.

**Supplemental Figure 5. Betulin restricts the glycolytic activities of HCC cells.** (A) The glucose uptake capacity, (B) LDH enzymatic activity, (C) ATP and (D) lactate productions, and ECAR and OCR measurements of MHCC97-H cells treated with indicated concentrations of Betulin (100μmol/L, 30μmol/L, 10μmol/L, 3μmol/L, 1μmol/L, 0.3μmol/L or 0.1μmol/L) for 48 hours.

**Supplemental Figure 6. Betulin restricts the lipid and glucose metabolism-related and EMT-related gene expression.** MHCC97-H cells treated with indicated concentrations of Betulin (100μmol/L, 30μmol/L, 10μmol/L, 3μmol/L, 1μmol/L, 0.3μmol/L or 0.1μmol/L) for 48 hours. Next, cells were harvested for quantitative RT-PCR.

**Supplemental Figure 7 SREBP-1 knock-down synergizes Sorafenib-mediated restriction of HCC cell migration.** MHCC97-H cells transfected with control or SREBP-1 siRNAs were treated with vehicle control or Sorafenib at *IC_50_* concentration for 48 hours. Then Transwell experiment was performed.

**Supplemental Figure 8. Betulin inhibits MHCC97-H *s.c.* tumor growth.** (A) MHCC97-H cells were injected into nude mice subcutaneously. At day 6, when *s.c.* tumors established, mice started receiving vehicle control or indicated concentrations of Betulin via orally every other day for 10 times. At day 21, mice were sacrificed and tumors were obtained (N=10). (B) Quantitative results of tumor volume and tumor weight from (A).

**Supplemental Figure 9. Betulin inhibits the glycolytic activities of HCC cells *in vivo*.** (A) The inhibition rate of tumor growth, lactate and ATP productions, LDH activity and glucose uptake of tumor cells isolated from *s.c.* HCC tumors from Supplemental Figure 8. (B) The quantified data from (A). (C) The isolated HCC cells from each group prepared for quantitative RT-PCR. Gene expression was calculated and shown by heatmap.

**Supplemental Figure 10. SREBP-1 overexpression overcomes Sorafenib’s effect on HCC *s.c.* tumor growth.** (A) MHCC97-H *s.c.* tumors were established using MHCC97-L cells transfected with with empty or SREBP-1 expressing vectors. MHCC97-L *s.c.* tumor bearing mice received vehicle control, or Sorafenib at *IC_50_* concentration; or 2 mg/kg Betulin or Sorafenib + Betulin orally every other day for 10 times. At day 21 post-treatment, mice were sacrificed and tumors were obtained (N=10). (B) Quantitative results of tumor volume and tumor weight from (A). (C) Western blotting of SREBP-1 and apoptosis and proliferation associated proteins in tumors from (A). β-actin was used as internal control.

**Supplemental Figure 11. SREBP-1 knock-down synergizes Sorafenib’s effect on HCC *in situ* growth in mouse liver.** (A) MHCC97-H *in situ* tumors were established using MHCC97-H cells transfected with control or SREBP-1 siRNAs, or SREBP-1 siRNAs plus SREBP-1^mut^ expressing vector. The *in situ* xenograft tumors were established by injected MHCC97-H cells directly into mouse livers. bearing mice received vehicle control, or Sorafenib at *IC_50_* concentration; or 2 mg/kg Betulin or Sorafenib + Betulin orally every other day for 10 times. At day 21 post-treatment, mice were scanned by *in vivo* small animals MicroPET imaging (N=10). (B) Representative Quantification results of tumor nodules from each treatment groups.

**Supplemental Figure 12. SREBP-1 overexpression overcomes Sorafenib’s effect on HCC *in situ* growth in mouse liver.** (A) MHCC97-L *in situ* tumors were established using MHCC97-H transfected with with empty or SREBP-1 expressing vectors. Tumor bearing mice received vehicle control, or Sorafenib at *IC_50_* concentration; or 2 mg/kg Betulin or Sorafenib + Betulin orally every other day for 10 times. At day 21 post-treatment, mice were scanned by *in vivo* small animals MicroPET imaging (N=10). (B) Representative Quantification results of tumor nodules from each treatment groups.
